# Algal magnetic nickel oxide nanocatalyst in accelerated synthesis of pyridopyrimidine derivatives

**DOI:** 10.1038/s41598-021-85832-z

**Published:** 2021-03-18

**Authors:** Javad Moavi, Foad Buazar, Mohammad Hosein Sayahi

**Affiliations:** 1 Department of Marine Chemistry, Khorramshahr University of Marine Science and Technology, P.O. Box 669, Khorramshahr, Iran; 2 Department of Chemistry, Payame Noor University, P.O. Box 19395-3697, Tehran, Iran

**Keywords:** Biosynthesis, Chemistry, Catalyst synthesis, Catalytic mechanisms, Heterogeneous catalysis

## Abstract

This research presents a novel biological route for the biosynthesis of nickel oxide nanoparticles (NiO NPs) using marine macroalgae extract as a reducing and coating agent under optimized synthesis conditions. XRD and TEM analyses revealed that phytosynthesized NiO NPs are crystalline in nature with a spherical shape having a mean particle size of 32.64 nm. TGA results indicated the presence of marine-derived organic constituents on the surface of NiO NPs. It is found that biogenic NiO NPs with BET surface area of 45.59 m^2^g^−1^ is a highly efficient catalyst for benign one-pot preparation of pyridopyrimidine derivatives using aqueous reaction conditions. This environmentally friendly procedure takes considerable advantages of shorter reaction times, excellent product yields (up to 96%), magnetically viable nanocatalyst (7 runs), low catalyst loadings, and free toxic chemical reagents.

## Introduction

In recent years, the marine realm contribution to the production of versatile nanoparticles has undergone significant growth across international research communities since its economic, rapid, and consequently, environmental procedures render a viable template for the biosynthetic route^1,2^. Marine organisms (e.g. algae, yeast, bacteria, fungal species) owing to possessing a broad variety of electron-rich phytochemicals such as polyphenols, carbohydrates, polyols, alkaloids, proteins, polysaccharides, peptides, and amino acids, establish a sing-step and comprehensive platform for the simultaneous reduction of metal cations and stabilization of biofabricated nanoparticles^3^. In contrast to commonly employed physical and chemical approaches, the sheer growing demand for uses of green processes presumably due to environmental compatibility, simplicity, and the absence of detrimental chemical reagents^4^.

Algae are highly diversified naturally occurring microbes and inhabitants of various aquatic environments such as marine- and fresh-water. These microorganisms are commercially and biologically of great importance since they contain valuable phytoconstituents that address the global market needs through virtual engagement in commercial products, for instance, cosmetics, biofuel, and drugs. In the aspect of biosynthesis, marine algae are known as “bionanofactory”, and hence, different nanoscale materials including alumina, zinc oxide, gold, have been synthesized through miscellaneous algae as a competent green synthesis method^5–7^.

Catalyst is a focal point in enormous chemical reactions and has been gaining a surge of popularity particularly in academic and industrial synthetic reactions areas over the past decades. Interestingly, convergence between nanoscience and chemistry led to the advent of nanocatalysts in which as a result of immense surface areas and expansive catalytic capabilities, have attracted great attention of many organic chemists in a myriad of catalyzed-reactions^8,9^. Apart from using a trace amount of catalyst, nanocatalysis processes dramatically enhance the contact between the active component of the catalyst and reactants and therefore increase the rate and yield of chemical reactions^10,11^. A vast majority of the heterogeneous and homogenous catalysts are pertinent to transition metal nanoparticles due to their unprecedented physicochemical properties^12–15^. Among these tremendous efforts, nickel oxide nanoparticles have been developed as an efficient catalyst in the chemical synthesis of a wide range of valuable organic compounds such as spiro and condensed indole derivatives^16^, aromatic heterocycle^17^, 5-substituted 1 h-tetrazoles^18^, quinolines^19^, spirooxindoles ^20^, polyhydroquinolines, and sulfoxidation^21^. There are a variety of green methods that have been addressed in the construction of the nickel oxide nanoparticles (NiO NPs) for various application^22^. It is worth mention that the plant-assisted bioreduction strategy for NiO NPs fabrication has received glob attention as a renewable and sustainable supplier^23–25^.

Multicomponent Reactions (MCRs) are propitious condensation reactions^26,27^, in which integrate moieties of several starting materials to produce a desirable product^28,29^. This efficient chemical synthesis toolbox is of great interest due to high selectivity, bond-construction efficiency, and sustainability in providing complex molecules with promoted diversity^12,15,30^. Moreover, MCR are considered as a combinatorial synthesis approach in which a chemical reaction of all materials is occurred in one pot in a single step fashion producing a wide variety of organic compounds with relatively strict avoidance of waste formation^31–34^.

**Scheme 1:**
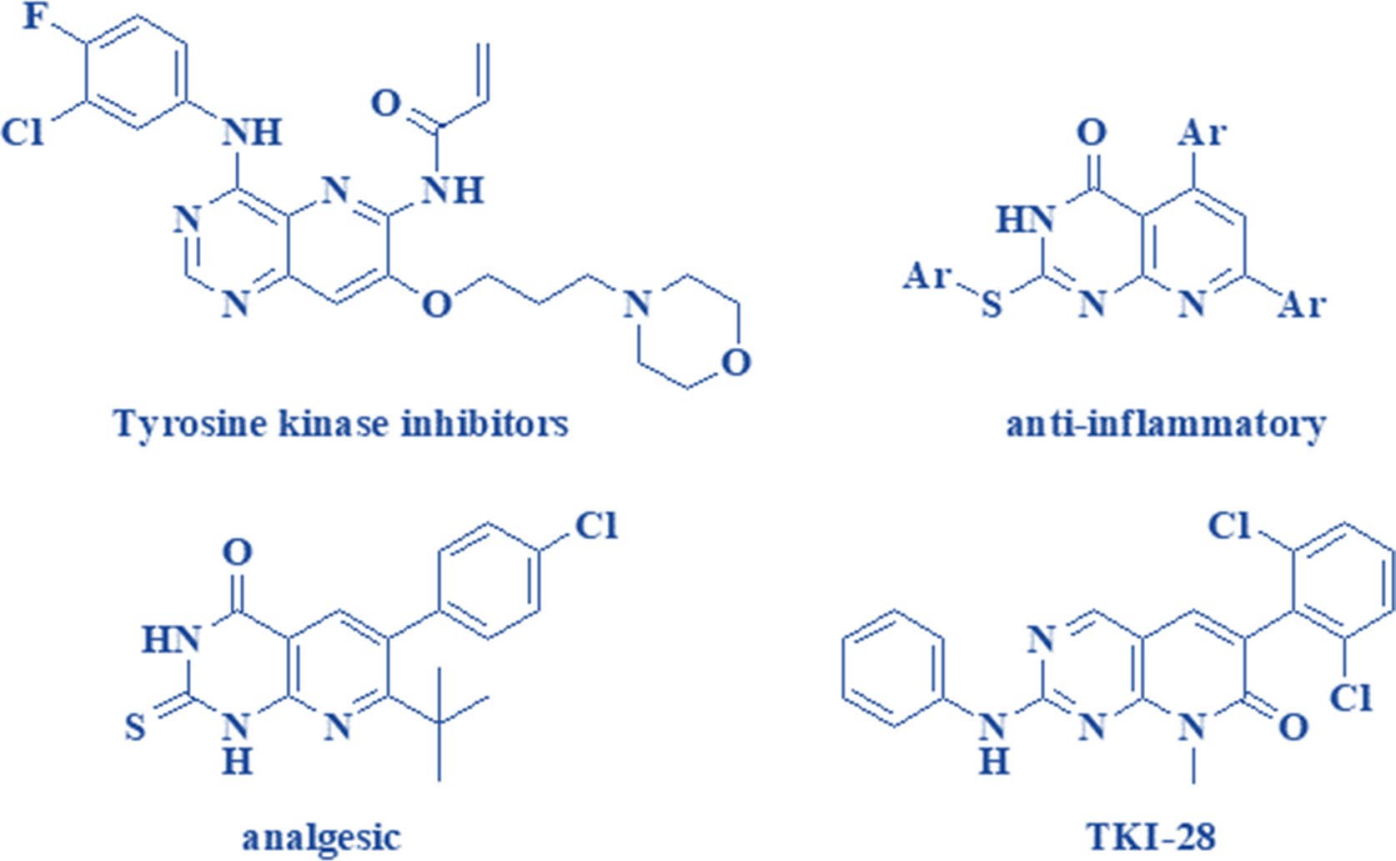
Selected pyridopyrimidine compounds with pharmaceutical properties^27^.

Pyridopyrimidines and their fused heterocycles derivatives are of paramount importance due to their significant biological and pharmacological activities. Moreover, owing to the involvement of pyridopyrimidine skeleton in some essential medicinal drugs, they hold great promise in the area of pharmaceutical science. Compounds possessing the pyrido[3,2-d]pyrimidines scaffold demonstrate a broad spectrum of biological activities such as antiviral^35,36^, antimicrobial^37^, antihypertensive, anti-tumor^38^, antihistaminic^39^, antimalarial, potent inhibitor of protein kinases^40^, treatment of diarrhea^41^, anti-inflammatory and analgesic activity^42^, along with other medicinal applications (Scheme 1)^43^. Due to their large variety of characteristics, there has been a growing demand in the design and production of pyridopyrimidine nucleus derivatives in particular those derived from proactive biological procedures.

In this study, proceeding our interest toward green nanoparticle synthesis and application^44^, we report a novel biosynthetic method to produce NiO NPs through electron-rich marine algae extract by optimizing these reaction conditions. Afterward, the catalytic efficiency of marine-assisted NiO NPs was explored in the eco-friendly synthesis of pyridopyrimidine derivatives by one-pot three-component elaborated in green conditions. The entire synthetic reaction was performed in water as a highly desirable solvent where increases the environmental impact and economical perspective of the designed protocol^45^. Based on the literature survey, this is the new report on the use of marine algae-assisted NiO NPs as a heterogenetic catalyst in organic synthesis of a library of pyridopyrimidine heterocyclic compounds through newly four-component condensation.

## Results and discussion

### UV–visible spectroscopy analysis

**Figure 1:**
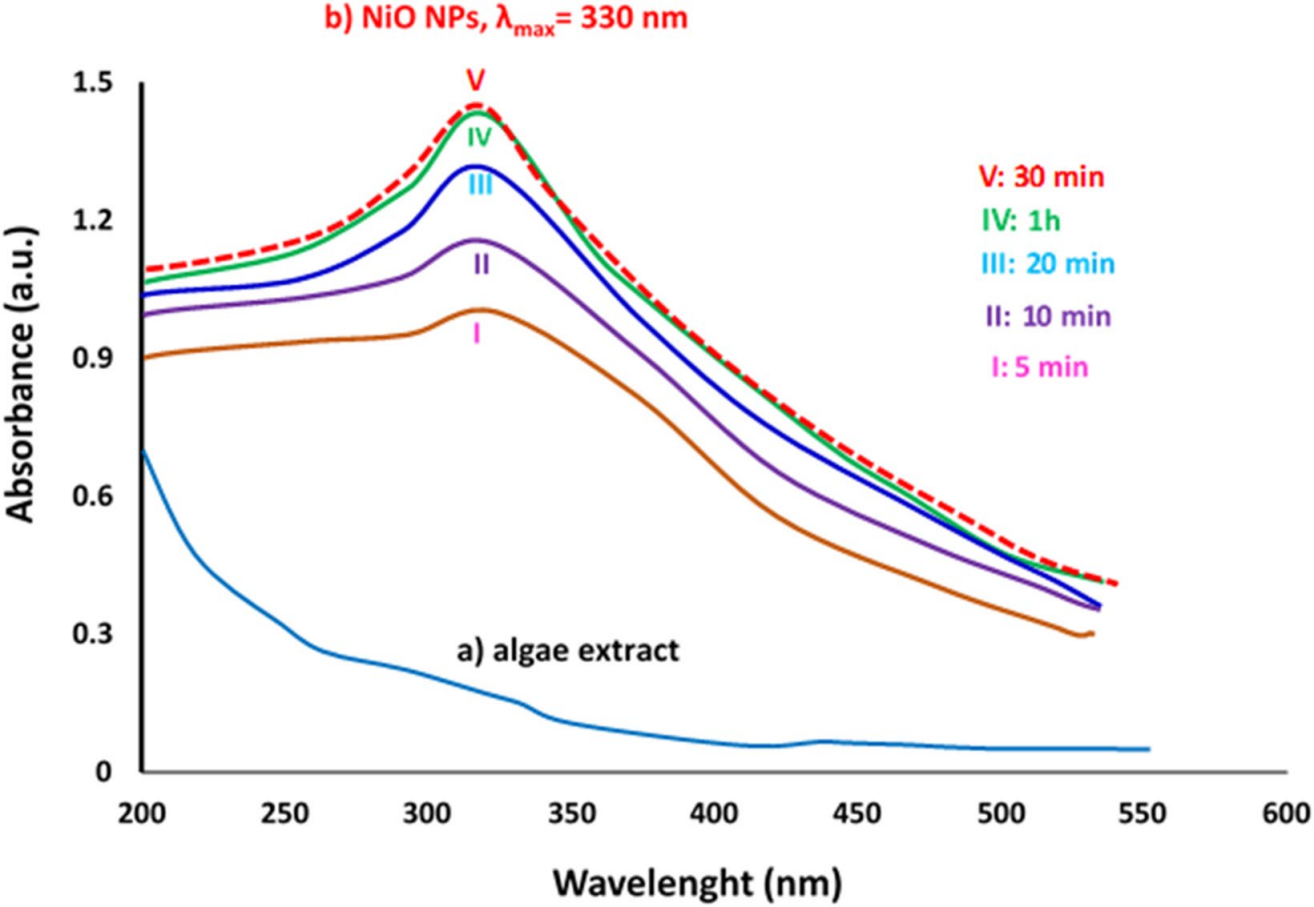
UV–visible spectra of (**a**) organic seaweed extract, and (**b**) optimization of biogenic NiO NPs as a function of time.

The initial evidence of NiO NPs formation was revealed when the solution color changed from brown to dark green. Correspondingly, the UV–vis spectrum demonstrated a characteristic absorbance peak at 330 nm indicating biofabrication of NiO NPs in the extract of marine algae biomass (Fig. 1a, b). The completion of the reaction was monitored in UV visible spectra as a function of time (5 min, 10 min, 20 min, 30 min, and 1 h). The results show that the apex of the absorption peak intensity was observed at 30 min indicating the reduction of nickel ions to zero valance metallic nickel atom. Moreover, no sensible changes have been observed in peak position when the sample stored up to six months in the laboratory, indicating high stability of bioproduced NiO NPs in aqueous green media. Previous studies reported the optical absorption peak of biological NiO NPs in the range of 330–350 nm using green synthesis methods^24,25,46^ which are in good agreement with our results.

### XRD analysis

**Figure 2:**
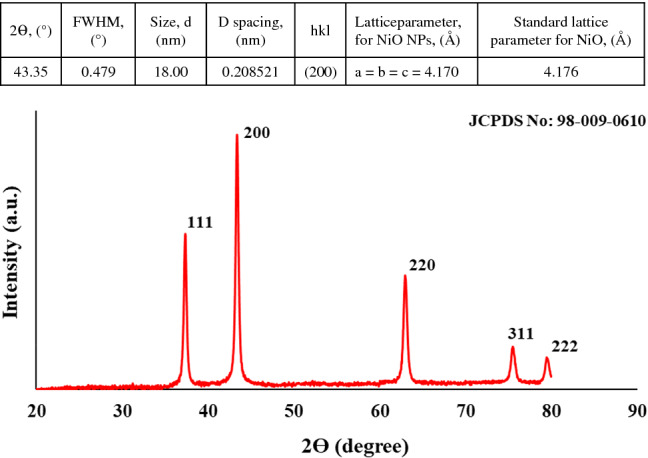
XRD pattern and crystal data of biosynthesized NiO NPs using red marine algae extract.

The phase structure of marine algae-mediated NiO NPs is further scrutinized by the X-ray diffraction technique. The crystal data (α = β = γ = 90°, lattice parameters, a = b = c = 4.1790 Å), prominent diffraction angles and miller indexes (hkl) at 37.45°(111), 43.15°(200), 62.77°(220), 75.34°(311), and 79.64°(222), deduced from XRD pattern revealed that biogenic NiO NPs possess face center cubic (FCC) crystalline structure with FM3-M space group (Fig. 2)^47^. Owing to the nonappearance of impurity peaks, the biofabricated nanoparticles were highly pure in nature. In addition, our obtained data are well-matched with JCPDS No: 98–009-0610, showing similar results to literature reports concerning biosynthesized NiO NPs^48,49^. Based on the high intensity of Bragg peak at 43.35°(200), the calculated median crystallite size of NiO NPs was 18 nm using Debye–Scherrer’s formula.

### TEM characterization

**Figure 3:**
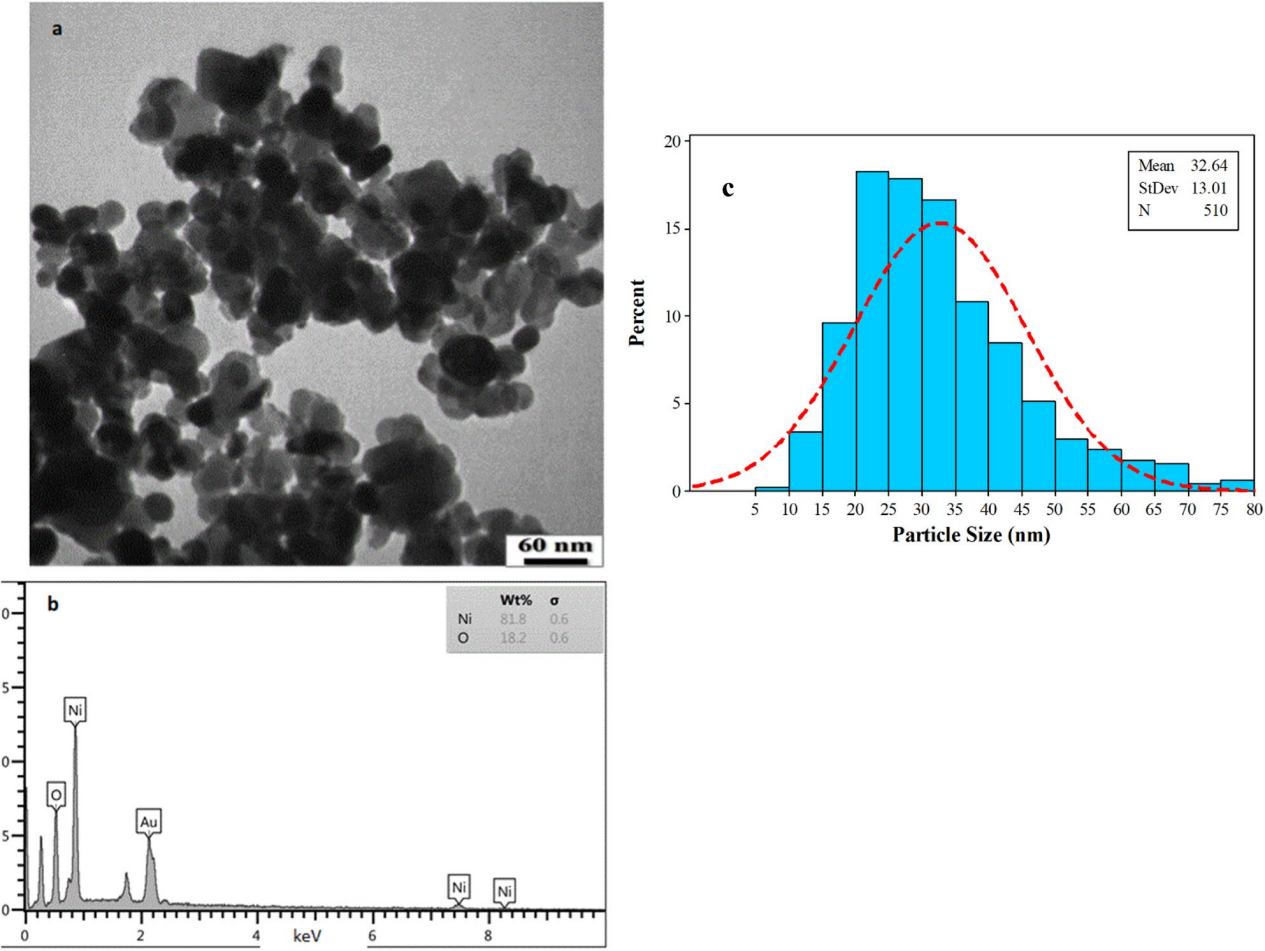
(**a**) The TEM image at 60.000 KX magnification, (**b**) EDX analysis, and (**c**) histogram of particle size distribution of algal NiO NPs.

The particle size and morphology and of NiO NPs were determined through the TEM analysis. TEM images confirmed that the vast majority of the particles were particles relatively non-spherical in shape, however, they appear in uniform and smooth morphology in the green synthesis platform as highlighted in Figure 3a. It can be noted that some regions of the sample appear darker in TEM image of bioprepared NiO NPs perhaps as a result of variation in thickness or high mass density of particles. Furthermore, the histogram of TEM image assay exhibited that biogenic NiO NPs possess a median diameter of 32.64 nm obtained through counting 510 particles as performed by ImageJ software (Figure 3c)^50^. EDX analysis confirmed the major components of the sample with nickel and oxygen atoms indicating the purity of biogenic Ni NPs (Figure 3b). The Au element detected in the EDX analysis was resulted from the gold-coated grid used for SEM specimen preparation.

### FTIR analysis

**Figure 4:**
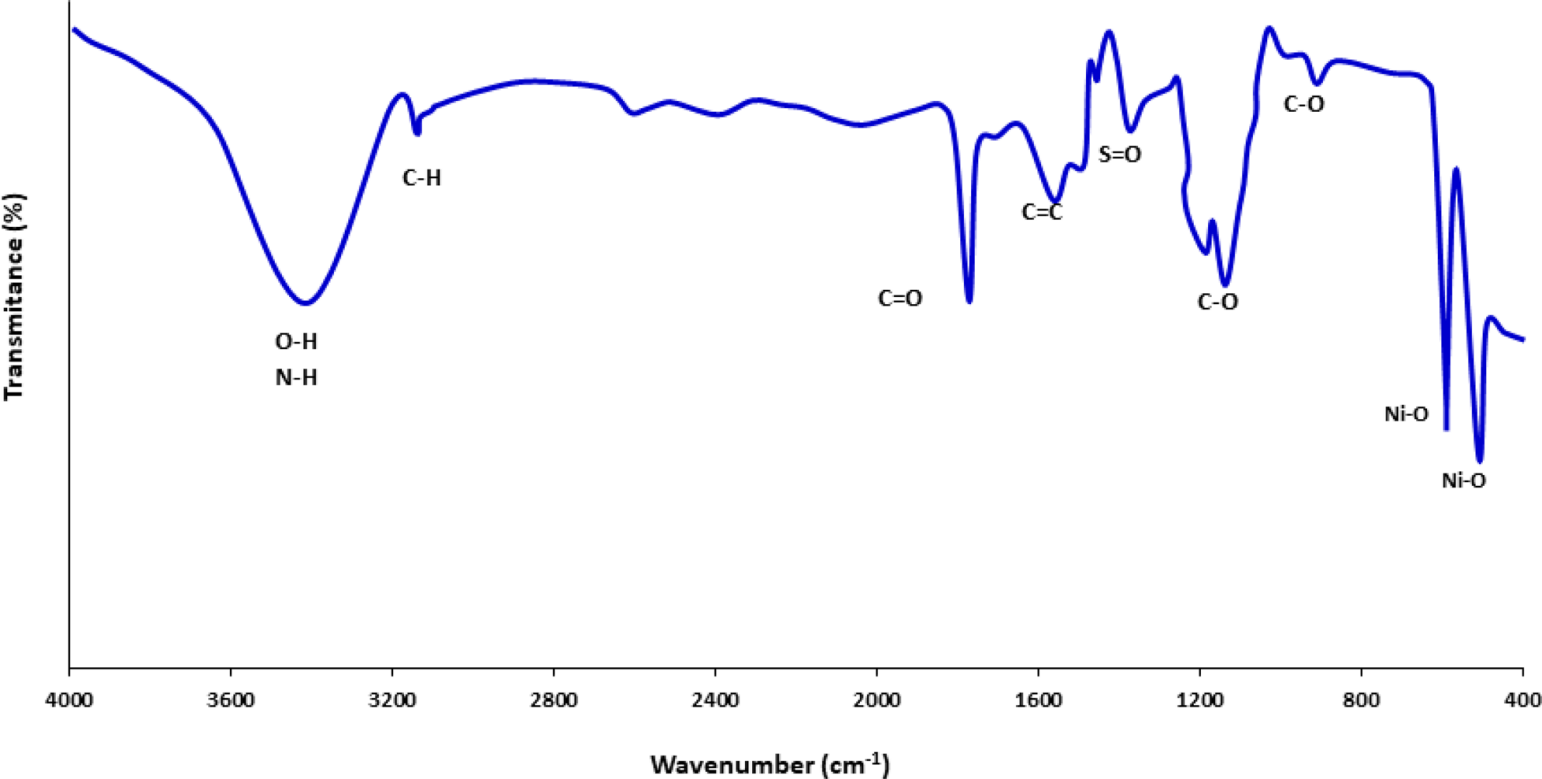
FTIR spectra of marine algae-mediated synthesized NiO NPs.

FTIR spectroscopy is a powerful tool to determine organic constituents qualitatively in seaweeds and plants^5^. The recorded spectrum presents a wide variety of prominent functional groups on the surface of biological NiO NPs as depicted in Fig. 4. The results of fingerprint regions of main functional groups on surface of fresh NiO NPs were also summarized in Table 1. The estimation of the class of organic moieties in the seaweeds is of great value since it would elucidate their role in the reduction and capping of freshly produced nanoparticles in the green medium. The strong wide band at 3411 cm^-1^ is characteristic of electron-rich N‒H and O‒H stretching vibrations, presenting amino acid and hydroxyl groups of polysaccharides. The weak C‒H stretching mode at 2945 cm^-1^ is attributed to the CH_2_ and CH_3_ groups of aliphatic compounds. The characteristic absorption peak around 1640 cm^-1^ is on account of the presence of C = O, pertaining to ester groups. The C = C absorption band representative of the lignin is observed at 1545 cm^-1^. The absorption band centered at 1322 cm^-1^ indicates S = O stretching of sulfated polysaccharide entity. Doublet symmetric C‒O vibration peaks appeared at 1030 and 1193 cm^-1^ may belong to ethers and glycosidic of carbohydrates. The advent of strong characteristic vibrations at 525 and 685 cm^-1^ indicates Ni–O bonds in the fingerprint region^24,48^. These results show the interaction of algal electron donor biomolecules with nickel cations maybe lead to the reduction as well as coating as-prepared NiO NPs. Apparently phytochemical presence in plant or microorganism extracts especially oxygen-contain biomolecules are acted through an oxidation–reduction mechanism, affording an appropriate reaction medium to generate eco-friendly nanomaterials^51^.

**Table 1:** FTIR characteristic absorption peaks of the engaged biomolecules in marine-mediated NiO NPs.

Entry	Functional group	Adsorption (cm^-1^)	References
1	O‒H, N‒H stretch	3411 cm^-1^	^52^
2	C‒H stretch	2945 cm^-1^	^53^
3	C = O stretch	1640 cm^-1^	^52^
4	C = C stretch	1545 cm^-1^	^54^
5	S = O stretch	1322 cm^-1^	^55^
6	C‒O stretch	1193 cm^-1^, 1030 cm^-1^	^52^
7	Ni‒O stretch	525 cm^-1^, 685 cm^-1^	^24,48^

### TGA investigation

**Figure. 5:**
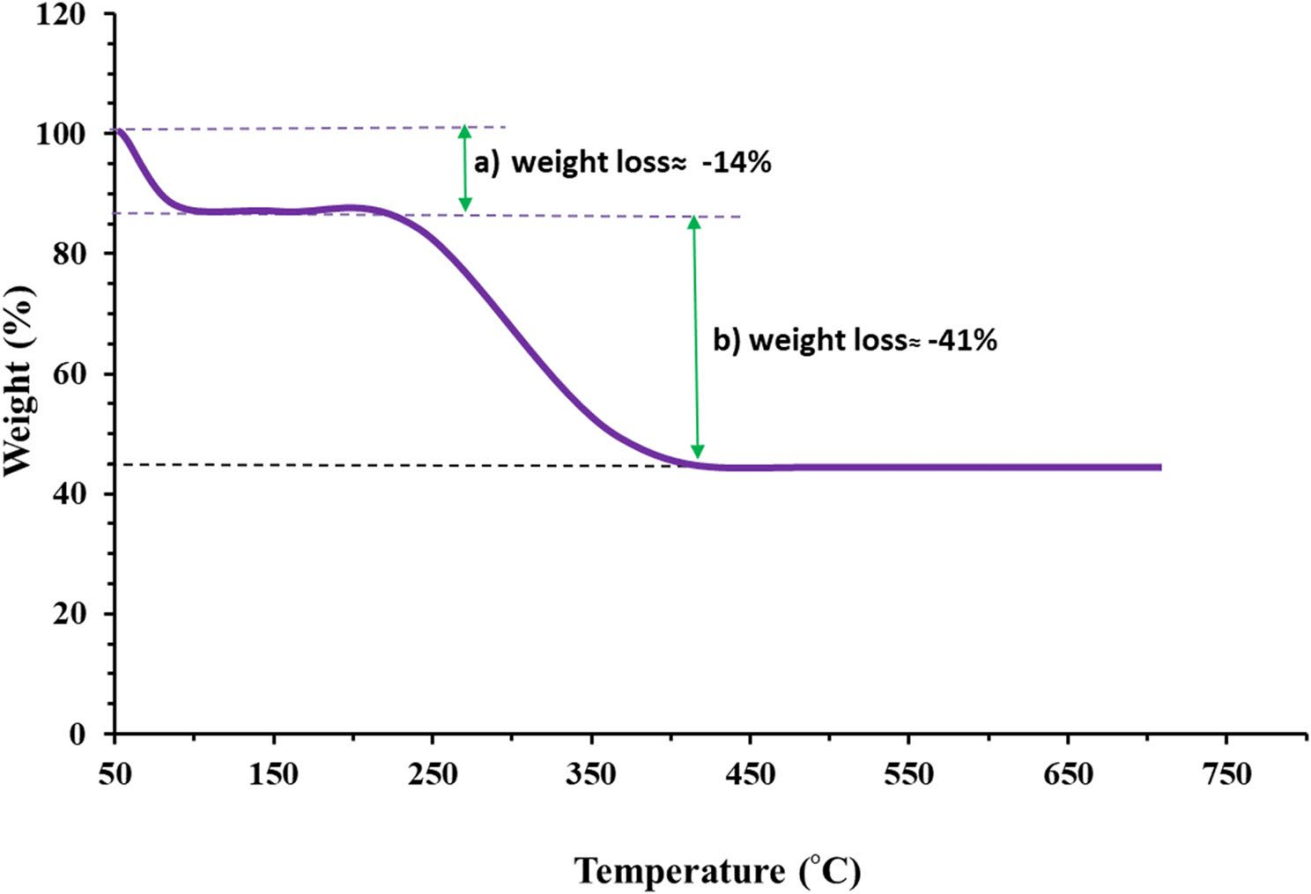
TGA curve of green NiO NPs using marine red algae extract.

The TGA technique is a powerful tool to explore green-assisted nanoparticles that encompassed organic biomolecules on their surface. The results of the TGA plot were depicted in Fig. 5. The first steady weight loss rate of 14% was occurred at the range of temperatures between 70 and 120 °C which is assigned to the vaporized H_2_O molecules absorbed on the surface of as-prepared NiO NPs. The second major loss with a proportional amount of 41% was observed at the temperature range of 220–420 °C most probably due to decomposing of marine-derived organic compounds that decorated the NiO NPs. Consequently, TGA curve findings are in good agreement with FTIR results fortifying the presence of propitious biofunctional groups which in turn could enhance the reduction of metallic nickel cations and coat the peripheral surface of freshly bioengineered nanoparticles.

### VSM properties of NiO NPs

**Figure 6:**
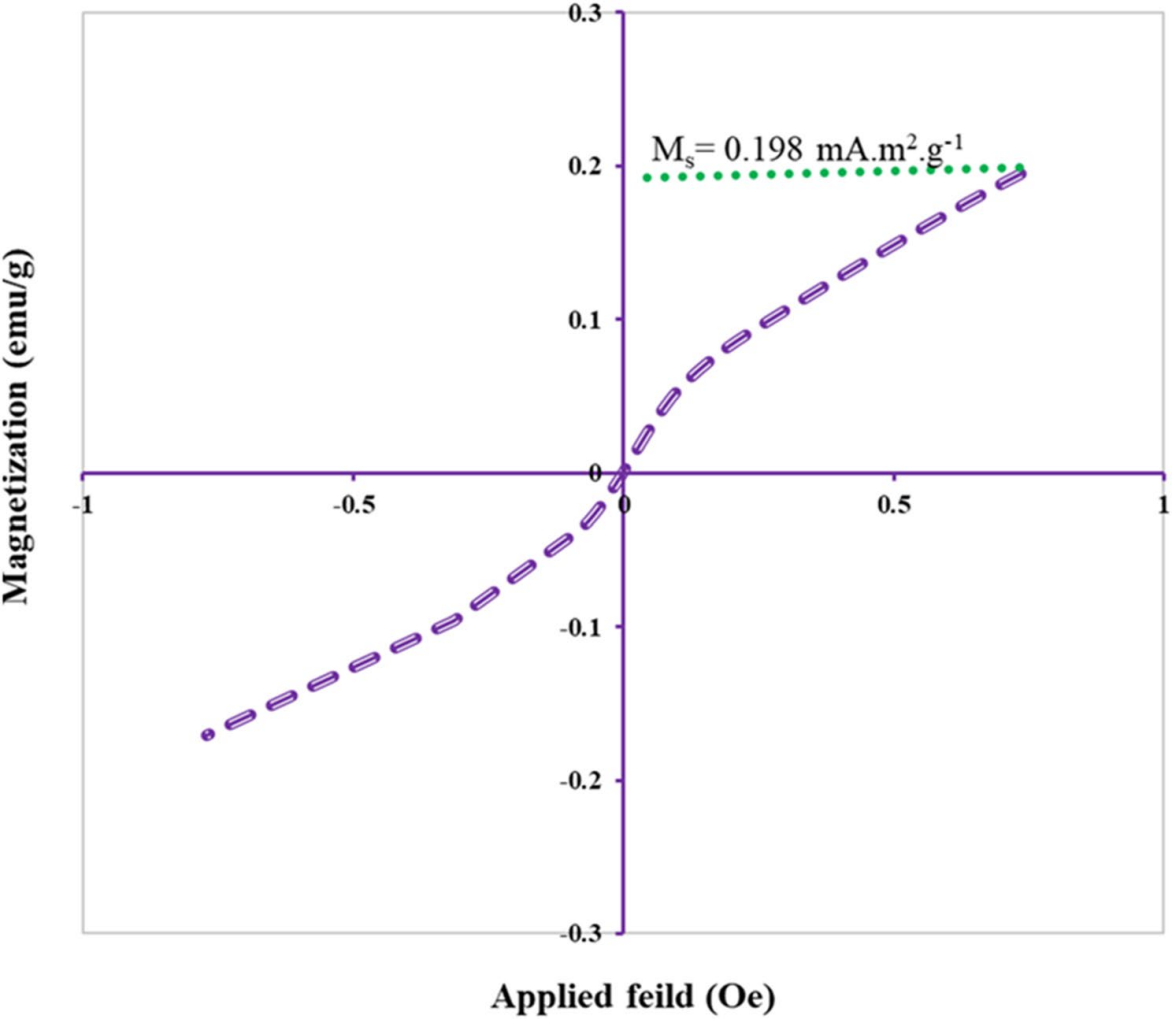
VSM magnetization curve of algal NiO NPs.

The vibrating sample magnetometer (VSM) analyzer was used to measure the probable magnetic amount of biologically formed NiO NPs. Figure 6 illustrates the magnetization M curve of green NiO NPs, after annealing, at fields of − 10 and + 10 Oersted. The M and H lines are not intersected in the entire curve indicating that coercivity (H_c_) and remanent magnetization (M_r_) were naught. As a result, algal NiO NPs could deem as superparamagnetic material. The saturation magnetization (M_s_) is the maximum value of magnetic induction and is size-dependent in which crystal lattice defects, inferior agglomeration state of the particles, size smallness, and higher surface-to-volume ratio would induce higher magnetic properties. Based on the VSM graph, Ms value of biogenic NiO NPs was measured as 0.198 emu/g which displays superior magnetization rather than reported plant-based NiO NPs^56^. It is well known that bulk grain-sized nickel oxide reveals antiferromagnetic susceptibility at room temperature^57^, whereas superparamagnetism is evidently a function of particle size^58^ for nanoparticles particularly below 20 nm as indicated for green NiO NPs.

### BET analysis

**Figure 7:**
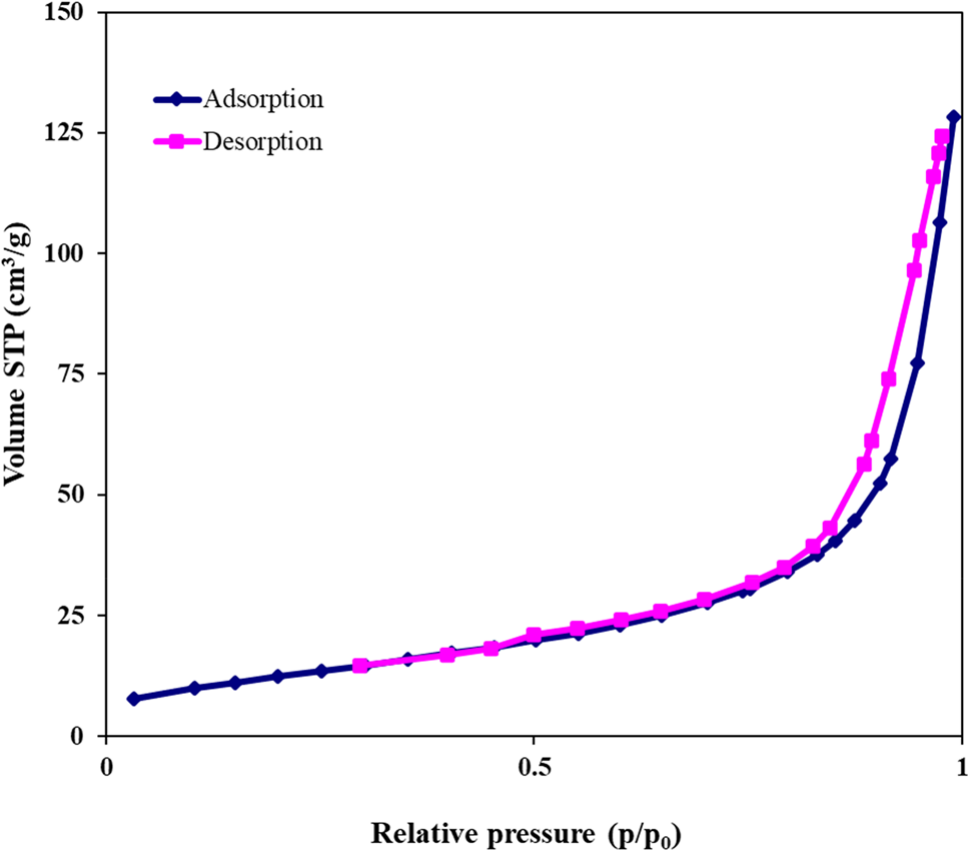
BET nitrogen adsorption/desorption isotherm of algal NiO nanocatalyst.

The BET curve of bio-assisted NiO NPs can be seen in Fig. 7. According to the result obtained from the BET isotherm measurement, the value of NiO NPs surface area, micropore volume, and average pore diameter were 45.59 m^2^g^−1^, 17.39 nm, 10.47 cm^3^g^−1^, respectively. As a result, the algae-mediated biosynthesized NiO NPs could deem as mesoporous material accelerating the catalyzed-organic synthesis reaction catalyzed-organic synthesis reactions^59^.

### Catalytic performance of biological NiO NPs

**Scheme 2:**
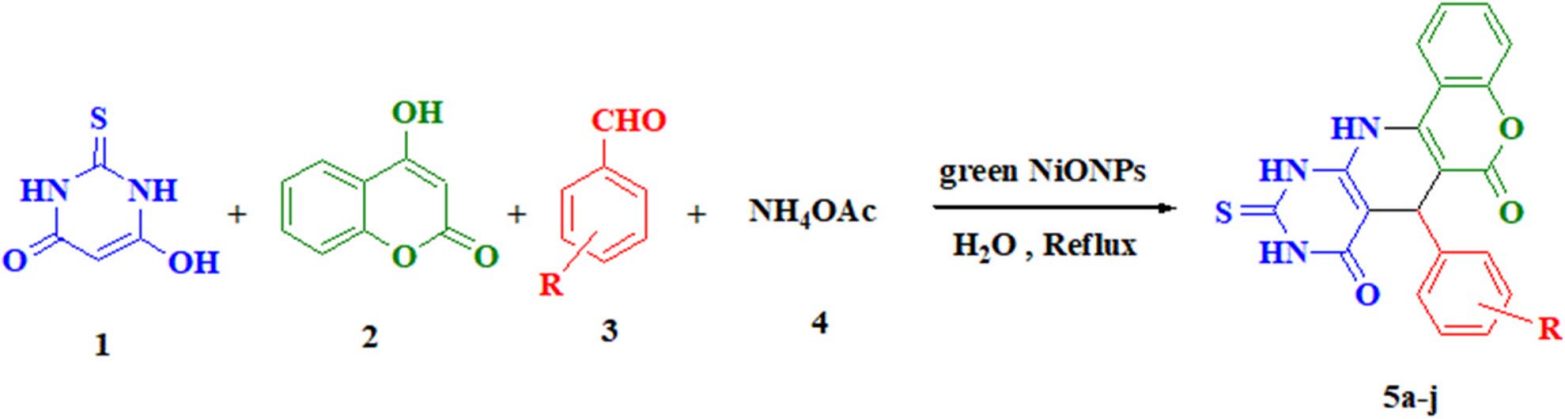
Synthesis of pyridopyrimidine derivatives **4a-j** catalyzed by biogenic NiO NPs via a four-component reaction.

In order to determine the optimal conditions of the reaction, we investigate the different amount of NiO catalyst required for the environmentally friendly synthesis of pyridopyrimidine derivatives using a straightforward four-component reaction approach of thiobarbituric acid **1**, 4-hydroxy coumarin **2**, aldehyde **3**, and ammonium acetate **4** as model substrates in water solvent condition (Scheme 2). It is found that the best yield of products (95%) was achieved when the molar concentration of nanoparticles reached 5% (0.004 mmol, 3 mg) (Table 2). It is noted that neither yield nor rate was revealed significant improvement by loading a larger amount of NiO nanocatalyst as illustrated in Table 2.

**Table 2:** Optimization of catalytic amount of green NiO NPs in the aqueous synthesis of **5g**.

Entry	Catalytic conc. (mol%)	Time (min)	Yield (%)
1	2	40	75
2	5	40	96
3	10	40	95
4	15	40	90
5	20	40	85

To assess the effectiveness of the catalytic activity of generated NiO NPs, a test reaction was explored in presence of various catalysts with respect to the reaction time of choice in an aqueous medium (Table 3). Generally, in the absence of a catalyst at a consistent temperature of 40 °C only offered trace yield of the expected products (entry 6). The results showed that the highest yield of products was obtained when biogenic NiO NPs contributed to the model reaction (entry 4). Based on TON (turnover number) and TOF (turnover frequency) results, the algal NiO NPs revealed greater stability and higher efficiency rather than other catalysts such as chemical bare NiO NPs (Table 3). Moreover, introducing chemical-produced NiO NPs (entry 2) increases the resultants yield rather than conventional catalysts (entries 1,3,5) in a shorter time whereas it produced lower yield and a longer reaction time rather than algal NiO NPs (entry 4). It is found that the occurrence of plant-derived phytochemical constituents on the surface of bio-assisted nanoparticles during the synthesis process, would tune the distinctive parameters of decorated nanomaterials such as morphology and particle-size distribution which in turn improve their catalytic properties rather than plain traditional catalysts^6^. For instance, the utilized biogenic NiO NPs in this study exhibited shorter reaction times and higher yields of products in comparison to poly (4-styrenesulfonic acid) mesoporous graphene oxide hybrid (PSSA-MGO) catalyst in the synthetic reaction of alike pyridopyrimidines^60^. As a result, the designed algal NiO NPs-catalyzed organic synthesis takes major advantages including excellent yields, short time, low cost, simplicity, green chemical reaction condition, and environmental sustainability.

**Table 3:** Comparison the efficacy of different catalysts in **5g** product preparation.

Entry	Catalyst	Time (min)	TON	TOF	Yield (%)
1	Bulk Ni oxide	240	12,200	50.83	61
2	Commercial NiO NPs	60	16,000	266.66	80
3	Acetic acid	120	10,000	83.33	50
4	Biogenic NiO NPs	40	19,200	480.0	96
5	HCl	90	6,000	66.67	30
6	Neat	Overnight	–	-	Trace

### Recyclability of the nanocatalyst

**Figure 8:**
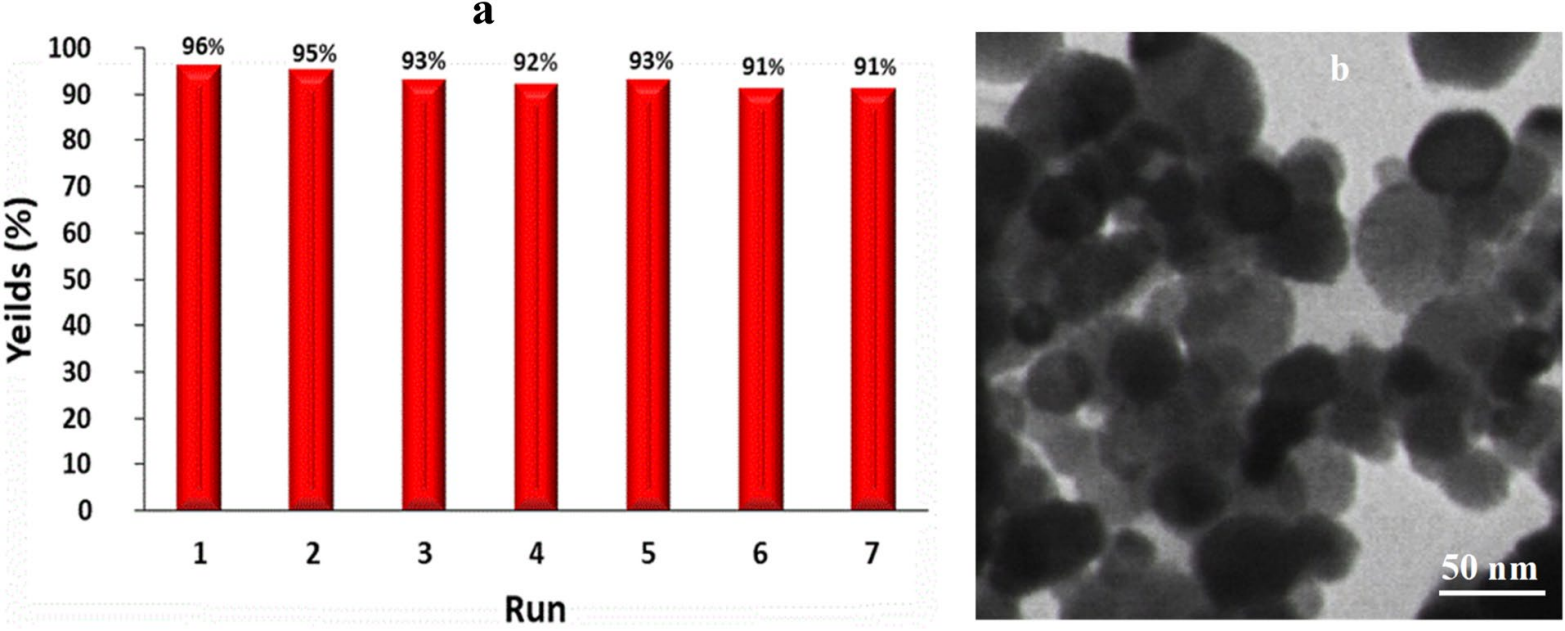
(**a**) Reusability and (**b**) TEM image of recovered algal NiO nanocatalyst in the synthesis of **5 g** in water.

The recycling of the catalyst is a pivotal point in the procedure of organic synthesis. In order to probe the reusability of biological magnetic NiO NPs; it was isolated from the reaction medium via an external magnet and washed several times with plenty of ethanol and water to achieve an unpolluted catalyst. It is found that no noticeable deterioration in the catalytic performance of algal NiO NPs was detected when it subjected to successive catalytic runs (up to seven) as depicted in Fig. 8a. The TEM image indicated that the morphology of the recovered nano-scaled NiO catalyst even after seven successive cycles relatively show no significant change after the reaction (Fig. 8b). In addition, the results of FTIR and EDX spectra exhibited no significant changes in surface chemistry and elemental composition indicating durability and stability reused biogenic NiO nanocatalyst (Fig. S2). Therefore, the desired pyridopyrimidine derivatives could furnish in the proficient yield in the presence of bioprepared NiO NPs.

### Scope and limits of biogenic NiO NPs efficiency

The generality of catalytic efficiency of NiO NPs was investigated using a library of miscellaneous functionalized organic aldehydes moieties under the optimized reaction conditions. Regardless of induced substituents, the produced pyridopyrimidine derivatives furnished in high product yields with appreciable purity, confirming the profound effect of green NiO NPs. Nevertheless, the variation of electron-donating and electron-withdrawing groups relatively change the yield of products. Moreover, the highest increase in the rate and product yield was acquired when the NO_2_ group was replaced at the para-position of aryl aldehyde producing the target product (**5g**) with a yield of 96% (Table 4, entry 7, Fig. S1).

**Table 4:** NiO NPs-catalyzed the synthesis of pyridopyrimidine derivatives^a^.

Entrya	R	Product	Time (min)	TONc	TOFd (min-1)	Yieldb (%)	MP (ºC) (Ref.)	
1	H		5a	45	17,600	391	88	235–237
								(236–238)^60^
2	2-Cl		5b	47	18,400	391.49	92	215–217
								(218–220)^60^
3	3-Cl		5c	50	18,000	360	90	220–222
								(219–220)^60^
4	4-Cl		5d	42	18,800	447.62	94	246–248
								(244–246)^60^
5	2,4-DiCl		5e	48	17,000	354.16	85	227–229
								(229–230)^60^
6	2-NO2		5f	50	17,000	360	90	236–238
								(235–237)^60^
7	4-NO2		5g	40	19,200	480	96	240–242
								(239–241)^60^
8	3-OCH3		5h	55	17,600	320	88	221–223
								(222–223)^60^
9	4-CN		5i	48	18,400	383.33	92	245–247
								(244–245)^60^
10	4-Br		5j	55	18,000	327.27	90	233–235
								(231–233)^60^

^a^Reaction conditions: 4-hydroxy coumarin (1 mmol), ammonium acetate (1 mmol), thiobarbituric acid (1 mmol), aromatic aldehyde (1 mmol), and NiO NPs (5% mol).

^b^Isolated yield.

^c^TON = turnover number = mol of product/ mole of catalyst.

^d^TOF = turnover frequency = mol of product/ mole of catalyst per min.

### Pyridopyrimidines synthesis mechanism

**Scheme 3:**
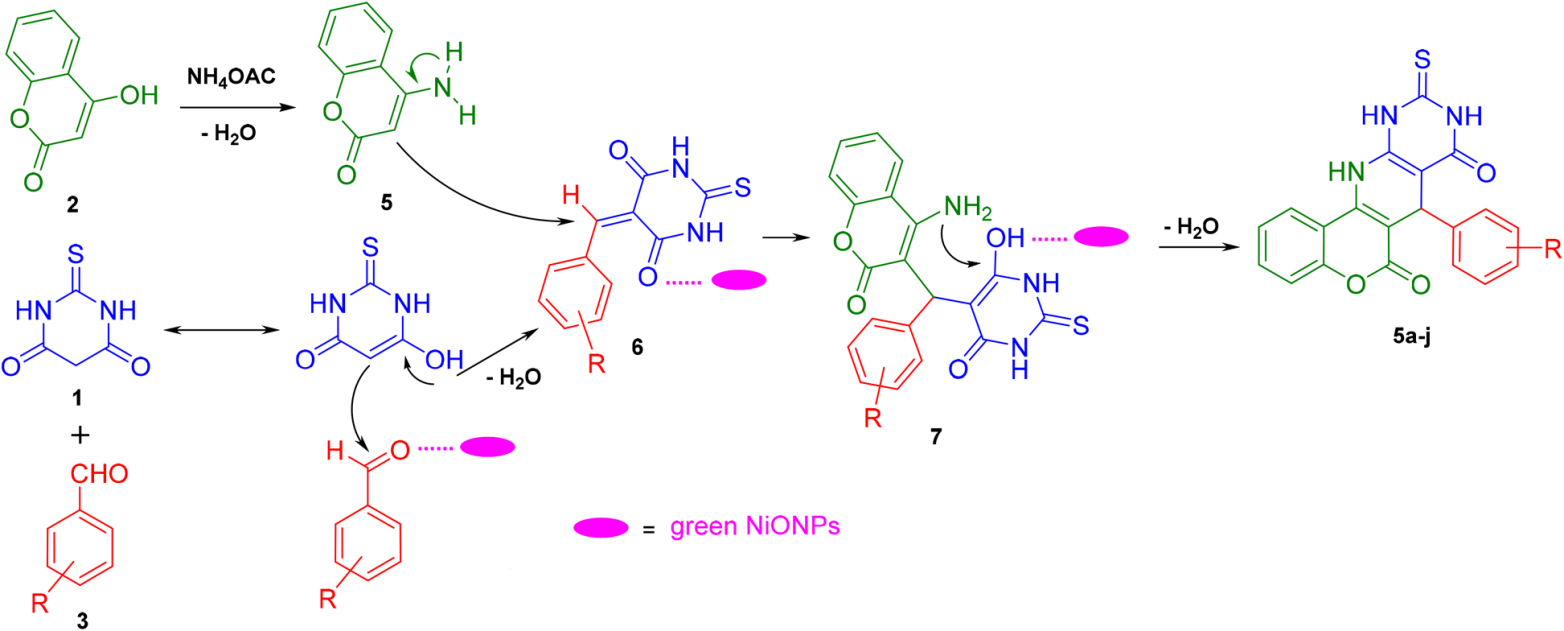
A proposed mechanism for the fabrication of pyridopyrimidine derivatives on the surface of biogenic NiO nanocatalyst.

According to the literature, a possible mechanism is proposed in Scheme 3 showing the pathway of catalyzed sequential multicomponent reactions. In the initial step, the enolic form of thiobarbituric acid (**1**) reacts with the catalyst-activated carbonyl of aryl aldehyde (**3**) through Knoevenagel condensation, to generate the α,β-unsaturated compound **6** followed by a dehydration reaction. In the next step, a reaction between 4-hydroxy coumarin (**2**) and in situ produced ammonia from ammonium acetate (**4**) is proposed to give 4-amino coumarin (**5**) at 100 °C. Then, via Michael addition of enamine (**5**) to an alpha, beta-unsaturated carbonyl acceptor of **6**, the intermediate **7** is formed. In the final step, the intramolecular ring cyclization occurs with an amino group attack on the carbonyl group with aid of NiO catalyst after lossing of H_2_O. As a result, the desired products **5a-l** are promoted within an aqueous medium at the appropriate time (Scheme 3). Apparently, introducing algal NiO NPs to the designed reaction would simultaneously boost the electrophilic character of the reactants and facilitate the attack of nucleophile groups owing to its acidic character. In addition, Lewis acid NiO nanocatalyst increases the generated intermediate stability and enhances the reactivity of organic materials as well^61,62^.

## Conclusion

We have demonstrated an efficient synthetic approach for the biosynthesis of nickel oxide nanoparticles using marine red algae extract as a novel natural source in the absence of hazardous reagents. It is found that algae-derived phytochemicals are effectively involved in bioreduction and coating as-prepared nanoparticles in benign conditions. The formation of NiO NPs was confirmed by using UV–vis, FTIR, XRD, TEM, TGA, BET, and EDX techniques. According to TON and TOF results the algal NiO NPs function as a highly robust catalyst for the one-pot facile preparation of pyridopyrimidine derivatives in water as a green solvent. The simple magnetic separation and high reusability of up to seven consecutive cycles further indicated the substantial catalytic activity of green NiO NPs. The sustainability, economic feasibility, and environmental compatibility of the proposed procedure could provide a broad range of uses in the processing and green synthesis of organic compounds.

### Materials and methods

All utilized chemicals in this study were obtained from Merck and used as received without any further purification. The formation of biofabricated NiO NPs was scrutinized using a UV–vis (Analytic Jena-Germany) spectrophotometer in the spectral range of 200–700 nm. To detect content variations of the functional groups, the FTIR spectra of the materials were recorded on Nicolet MAGNA-IR 550 spectrometer (Madison, WI, USA) by a KBr pellet. X-ray powder diffraction (XRD) diffractometer (Cu Ka, radiation, λ = 1.5405 Å) was run at a scanning speed of 2/min from 10 to 80 (2θ), examining the crystal structure of nanoparticles. To explore the magnetic properties of the nanoparticles, a vibrating sample magnetometer. (VSM, model BHV-55, Riken, Japan) the experiment was operated with a magnetic field up to 10 kOe. The morphology and particle size were obtained via transmission electron microscopy (TEM) operating with a Leo 912 AB at an accelerating voltage of 200 kV. For organic product characterization, melting points were gauged using the electrothermal IA9200 apparatus. Compositional analysis of the sample was carried out by X-ray energy dispersive spectroscopy (EDX). The progress of the reactions and assessment of the purity of the substrate was followed with TLC using silica gel SILG/UV 254 and 365 plates. To probe the surface of the sample, thermogravimetric analysis (TGA, PerkinElmer, Pyris 1, USA) technique was used. The surface area per mass of nanosample was measured using Brunauer–Emmett–Teller (BET) method. Finally, the specimens of red marine algae were gathered from the intertidal zone in coastal areas of Bushehr province, Iran, during low tide.

### Preparation of marine algae extract

In order to obtain a proper extract, in a 100 ml beaker 2 g of washed and under shade-dried powdered algae was soaked in 20 ml of distilled water under stirring at 180 rpm using a magnetic stirrer for 1 h then sonicated at 70 °C for 25 min. Further, the mixture was boiled for 10 min then cooled to room temperature and filtered through Whatman filter paper Grade No 1 (pore size: 11 µm). Eventually, the resultant crude extract was stored in an air-tight container and placed in the refrigerator for additional processing.

### Phyto-synthesis of NiO NPs

To synthesize nickel oxide nanoparticles, 10 ml of an aqueous solution of NiCl_2_·6H_2_O (0.01 M) was added to 90 ml of marine red algae extract. The reaction mixture containing red algae extract and nickel chloride hydrate solution was magnetically stirred at 800 rpm for 30 min at 60 °C to attain a homogeneous solution. After cooling down to room temperature, the purification of the resulting solution was fulfilled through continuous centrifugation at 12,000 rpm for 20 min with distilled water and ethanol to eliminate undesirable impurities followed by decanting. The obtained precipitate specimen was annealed at 450 °C for 90 min.

### Synthesis of pyridopyrimidines derivatives over NiO NPs catalyst

A mixture of ammonium acetate (**4**, 1 mmol) and 4-hydroxy coumarin (**2**,1 mmol) was refluxed in water (15 ml) for 30 min. Then, thiobarbituric acid (**1**,1 mmol), and aromatic aldehyde (**3**,1 mmol) in the presence of green NiO NPs (5 mol %, 0.003 g) as a heterogeneous catalyst were added and the reaction was stirred for further 10 min (Scheme 2). Upon completion of the reaction as monitored by TLC, the reaction solution was allowed to cool down to room temperature, subsequently, the 50 H_2_O was added to precipitate, filtered out and dried. The solid magnetic nanocatalyst was then separated by centrifugation and an external magnetic force, washed with 10 ml of pure ethanol, dried at 80 °C overnight, and reused. In order to afford uncontaminated products, the precipitate was boiled in 15 mL of EtOH for 5 min, and the final crystalline products were characterized using FTIR, elemental analysis, ^1^H and ^13^C NMR spectroscopic techniques (Figs. S1, see supplementary).

## Supplementary information

Supplementary information.
