# Supplementary material for: Algal magnetic nickel oxide nanocatalyst in accelerated synthesis of pyridopyrimidine derivatives
Source: Sci Rep. 2021 Mar 18;11:6296. doi: 10.1038/s41598-021-85832-z (PMC7973480; doi:10.1038/s41598-021-85832-z)
Supplement: Supplementary file 1 — Supplementary information. [file 41598_2021_85832_MOESM1_ESM.pdf]

Algal Magnetic Nickel Oxide Nanocatalyst in Accelerated Synthesis of Pyridopyrimidine Derivatives

Javad Moavi[a], Foad Buazar[a]*, Mohammad Hosein Sayahi[b]

[a]Department of Marine Chemistry, Khorramshahr University of Marine Science and Technology, P.O. Box 669, Khorramshahr, Iran

[b]Department of Chemistry, Payame Noor University, P.O. Box 19395-3697, Tehran, Iran

*Corresponding author: fb@kmsu.ac.ir (F. Buazar)
Tel.: (0098) 9161150684; Fax: (0098) 6153533322

***7-phenyl-10-thioxo-7,10,11,12-tetrahydro-6H-chromeno[3',4':5,6]pyrido[2,3-d]pyrimidine-6,8(9H)-dione* (5a)**

FT-IR (KBr, cm^{-1}): 3387, 3237, 1671, 1633, 1610, 1494; 1447, 1366, 1231, 1197, 1117, 757, 552, 537.

^{1}HNMR (DMSO-*d6*, 250 MHz) δ (ppm): 5.70 (s, CH-Ar, 1H), 7.10-7.71 (m, ArH, 8H), 7.94 (s, NH, 1H), 8.15 (m, ArH, 1H), 12.56 (s, NH, 2H).

^{13}CNMR (DMSO-*d6*, 62.5 MHz) δ(ppm): 34.9, 96.6, 98.4, 115.8, 118.6, 124.4, 124.8, 126.1, 127.3, 127.7, 129.7, 133.5, 134.3, 134.8, 139.6, 153.2, 156.7, 164.1, 167.4, 174.9.

MS, *m/z*: 375.07 (M^+); Anal. Calcd for $C_{20}H_{13}N_3O_3S$: C, 63.99; H, 3.49; N, 11.19; Found: C, 63.93; H, 3.55; N, 11.08.

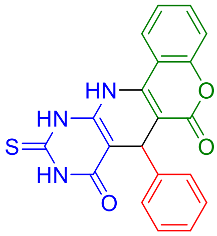

5a

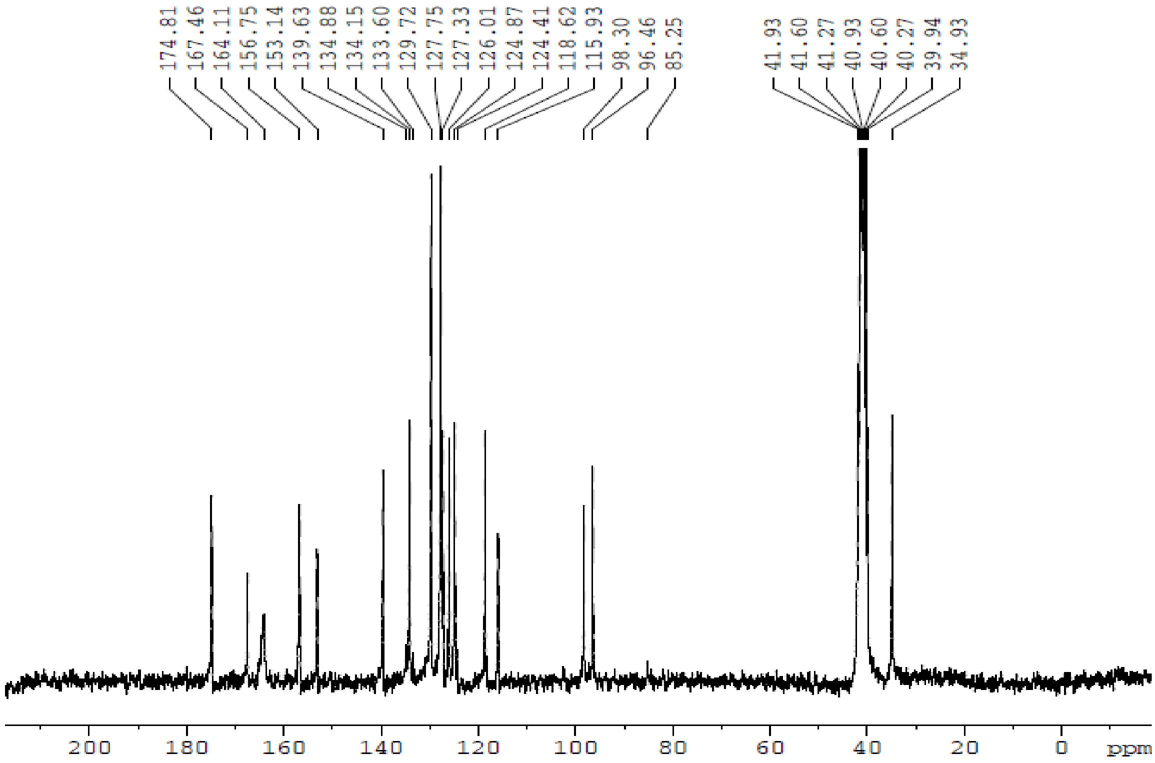
174.81
167.46
164.11
156.75
153.14
139.63
134.88
134.15
133.60
129.72
127.75
127.33
126.01
124.87
124.41
118.62
115.93
98.30
96.46
85.25
41.93
41.60
41.27
40.93
40.60
40.27
39.94
34.93
200
180
160
140
120
100
80
60
40
20
0
ppm

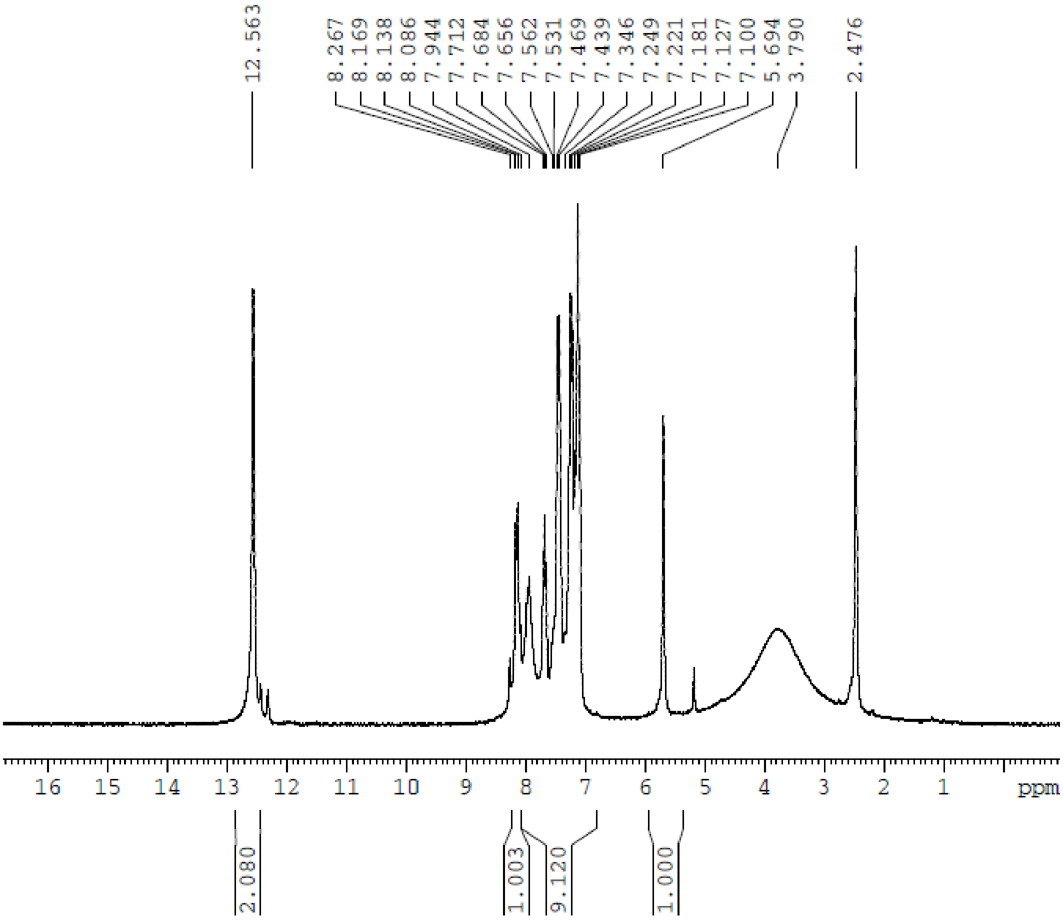
12.563
8.267
8.169
8.138
8.086
7.944
7.712
7.684
7.656
7.562
7.531
7.469
7.439
7.346
7.249
7.221
7.181
7.127
7.100
5.694
3.790
2.476
16
15
14
13
12
11
10
9
8
7
6
5
4
3
2
1
ppm
2.080
1.003
9.120
1.000

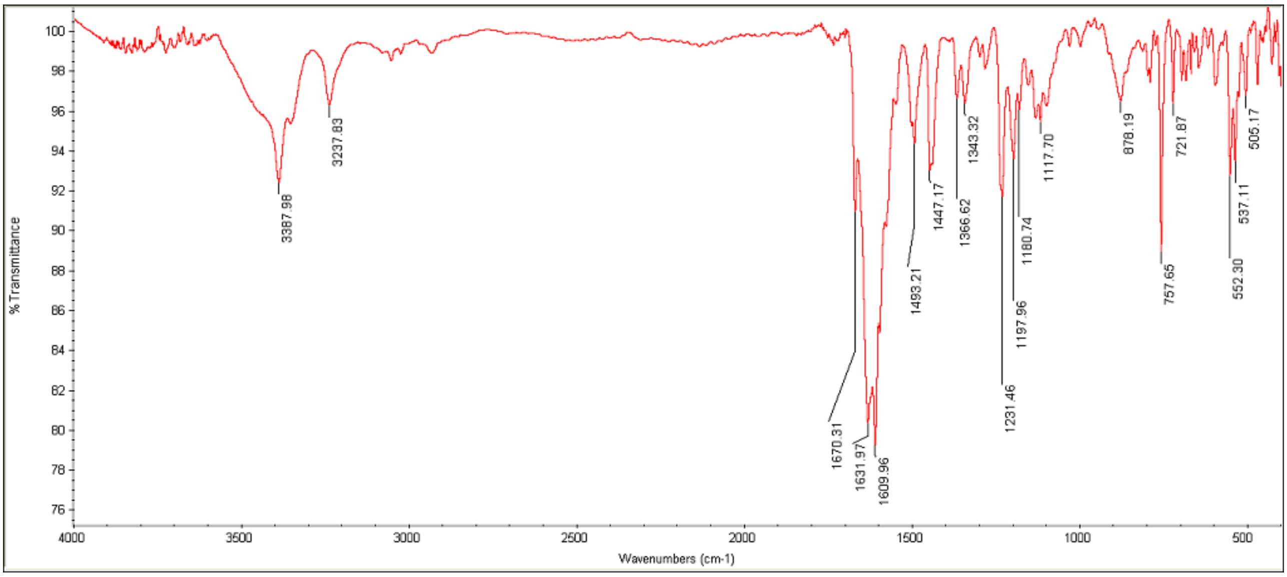

****

***7-(2-chlorophenyl)-10-thioxo-7,10,11,12-tetrahydro-6H-chromeno[3',4':5,6]pyrido[2,3-d]pyrimidine-6,8(9H)-dione* (5b)**

FT-IR (KBr, cm^{-1}): 3406, 3354, 3249, 2923, 1659, 1631, 1561, 1443, 1226, 1179, 1059, 918, 875, 760, 549, 536.

^{1}HNMR (DMSO-d_6, 250 MHz) δ (ppm): 5.70 (s, CH-Ar, 1H), 7.24-8.11 (m, ArH, 8H), 7.40 (s, NH, 1H), 12.40 (s, NH, 2H).

^{13}CNMR (DMSO-d_6, 62.5 MHz) δ (ppm): 35.3, 95.7, 97.5, 116.1, 118.5, 124.6, 125.7, 128.2, 129.4, 130.8, 131.3, 133.8, 134.2, 138.4, 153.3, 155.2, 163.8, 164.1, 166.2, 174.5.

MS, *m/z*: 409.03 (M^+); Anal. Calcd for $C_{20}H_{12}ClN_3O_3S$: C, 58.61; H, 2.95; N, 10.25; Found: C, 58.55; H, 2.90; N, 10.30.

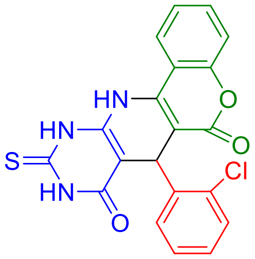

5b

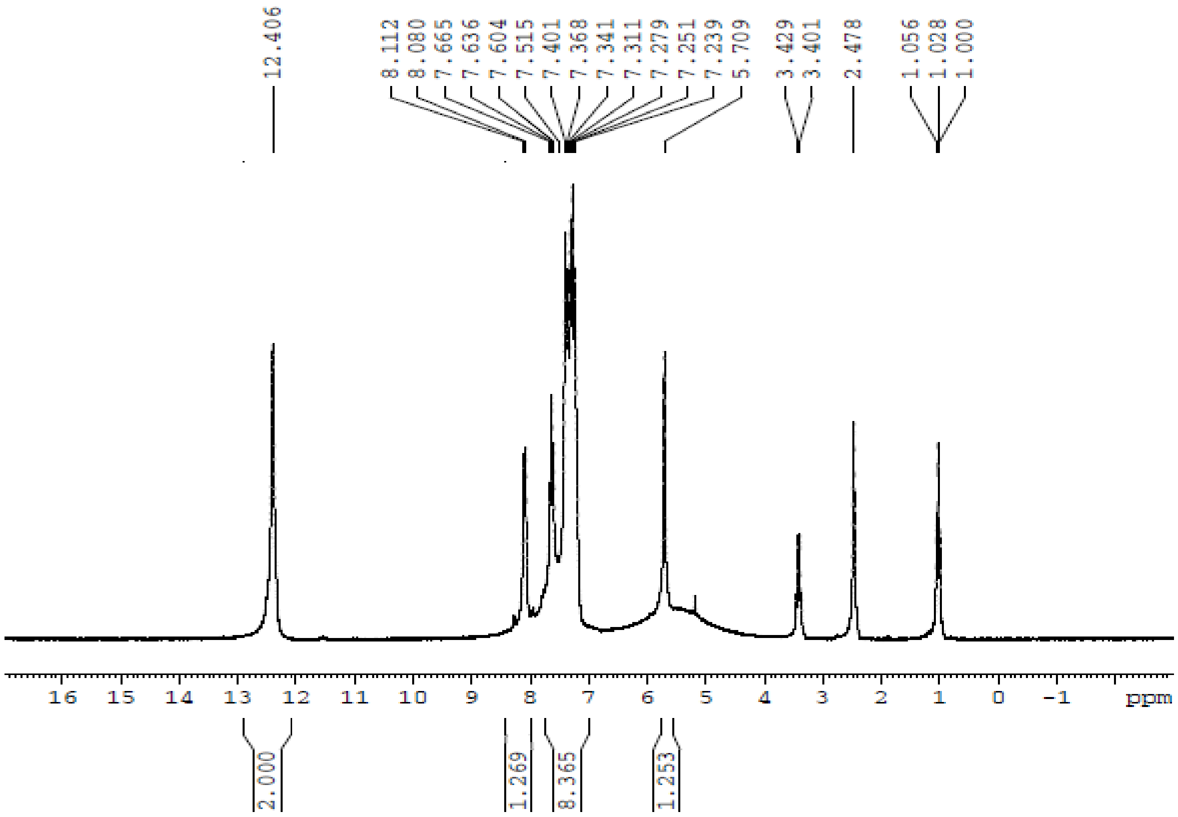
12.406
8.112
8.080
7.665
7.636
7.604
7.515
7.401
7.368
7.341
7.311
7.279
7.251
7.239
5.709
3.429
3.401
2.478
1.056
1.028
1.000
16 15 14 13 12 11 10 9 8 7 6 5 4 3 2 1 0 -1 ppm
2.000
1.269
8.365
1.253

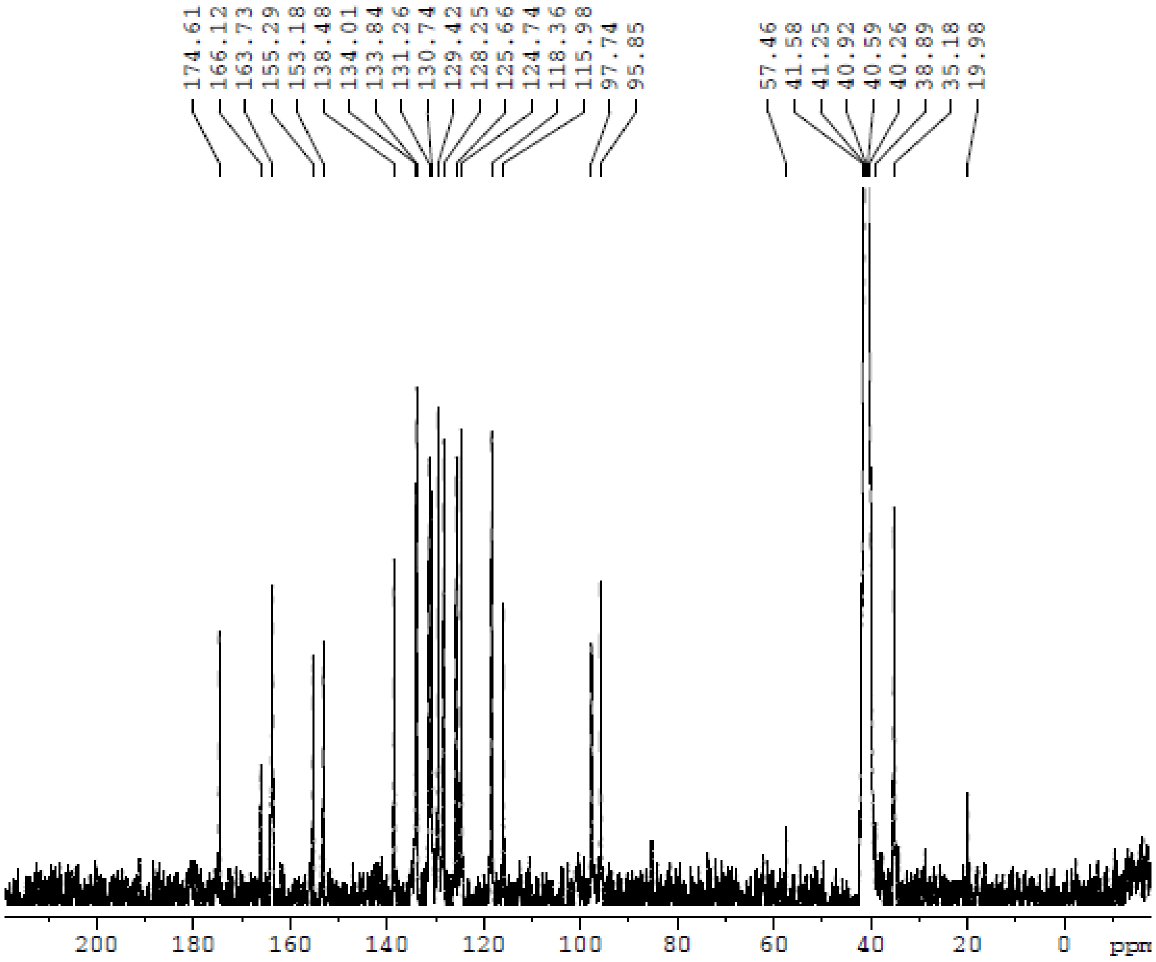
174.61
166.12
163.73
155.29
153.18
138.48
134.01
133.84
131.26
130.74
129.42
128.25
125.66
124.74
118.36
115.98
97.74
95.85
57.46
41.58
41.25
40.92
40.59
40.26
38.89
35.18
19.98
200 180 160 140 120 100 80 60 40 20 0 ppm

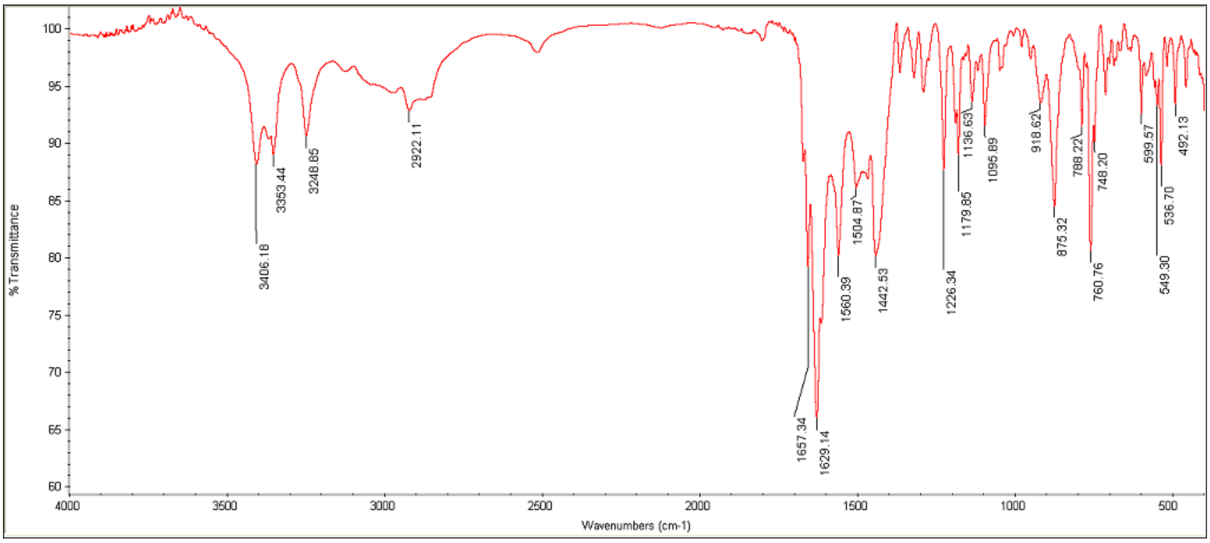

****

***7-(3-chlorophenyl)-10-thioxo-7,10,11,12-tetrahydro-6H-chromeno[3',4':5,6]pyrido[2,3-d]pyrimidine-6,8(9H)-dione* (5c)**

FT-IR (KBr, cm^{-1}): 3409, 3249, 2924, 1657, 1630, 1560, 1441, 1366, 1290, 1226, 1180, 1096, 875, 760, 712.

^{1}HNMR (DMSO-d_6, 250 MHz) δ (ppm): 5.71 (s, CH-Ar, 1H), 7.20-8.10 (m, ArH, 8H), 7.42 (s, NH, 1H), 12.3 (s, NH, 2H).

^{13}CNMR (DMSO-d_6, 62.5 MHz) δ (ppm): 35.4, 95.5, 97.8, 116.1, 118.4, 124.6, 125.7, 128.1, 129.3, 130.9, 131.1, 133.9, 134.1, 134.6, 138.7, 153.2, 155.3, 163.7, 166.1, 174.7.

MS, *m/z*: 409.03 (M^+); Anal. Calcd for $C_{20}H_{12}ClN_3O_3S$: C, 58.61; H, 2.95; N, 10.25; Found: C, 58.51; H, 2.88; N, 10.32.

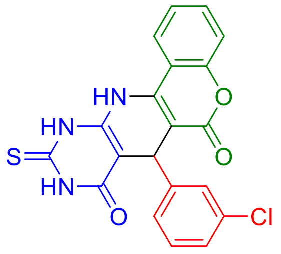

5c

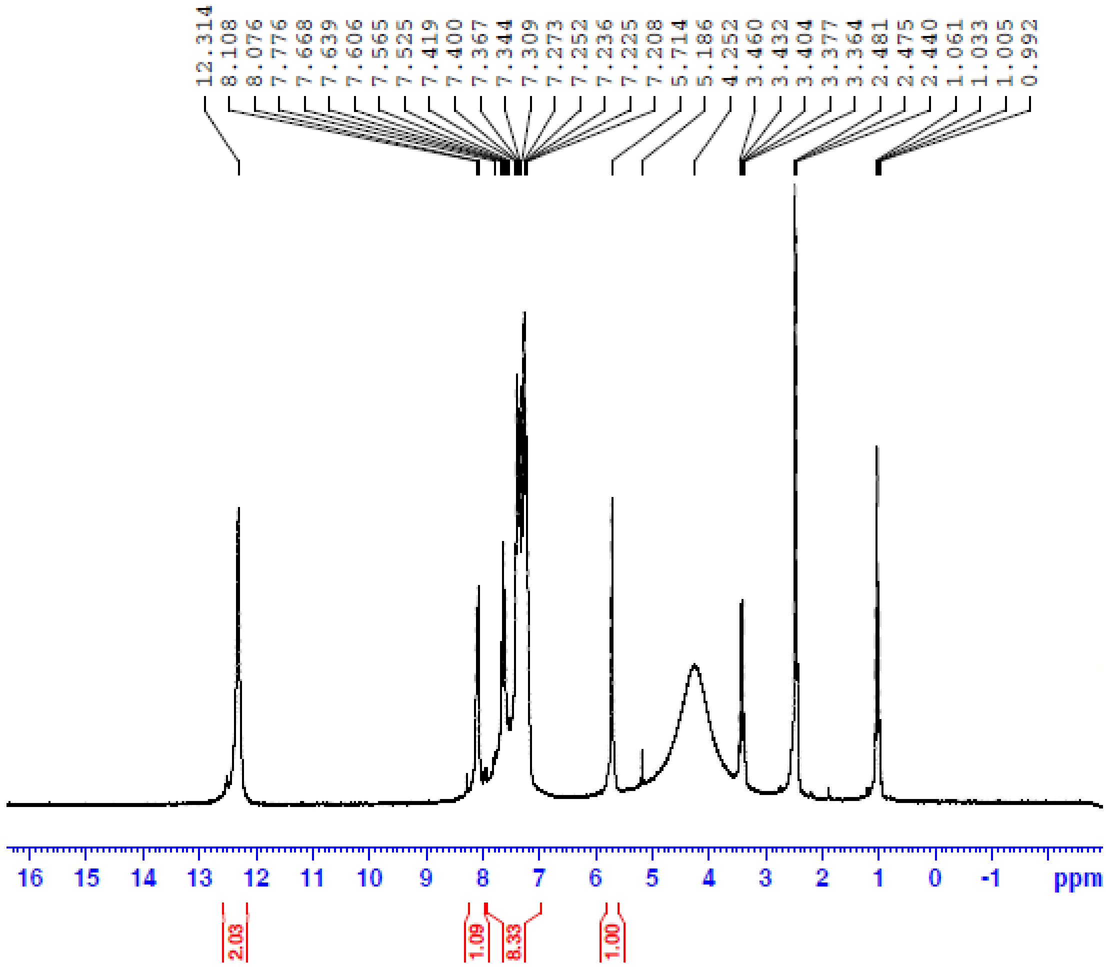
12.314
8.108
8.076
7.776
7.668
7.639
7.606
7.565
7.525
7.419
7.400
7.367
7.344
7.309
7.273
7.252
7.236
7.225
7.208
5.714
5.186
4.252
3.460
3.432
3.404
3.377
3.364
2.481
2.475
2.440
1.061
1.033
1.005
0.992
16 15 14 13 12 11 10 9 8 7 6 5 4 3 2 1 0 -1 ppm
2.03
1.09
8.33
1.00

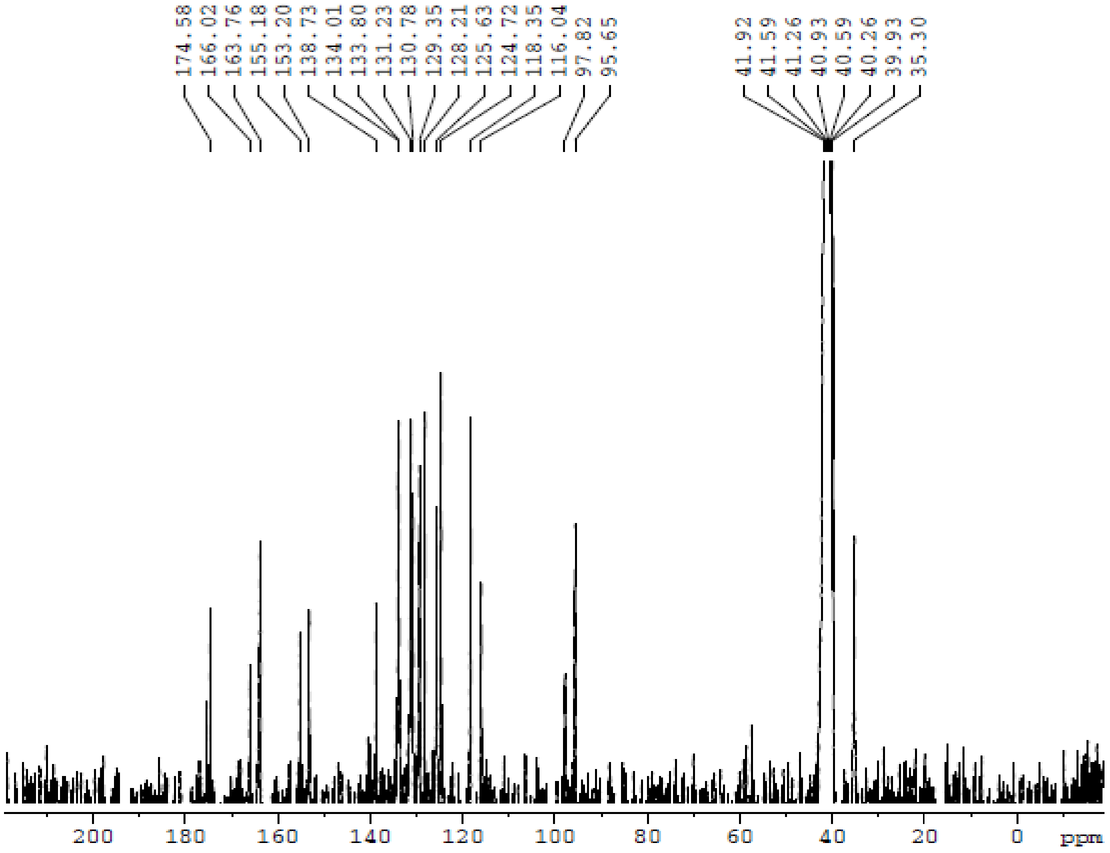
174.58
166.02
163.76
155.18
153.20
138.73
134.01
133.80
131.23
130.78
129.35
128.21
125.63
124.72
118.35
116.04
97.82
95.65
41.92
41.59
41.26
40.93
40.59
40.26
39.93
35.30
200 180 160 140 120 100 80 60 40 20 0 ppm

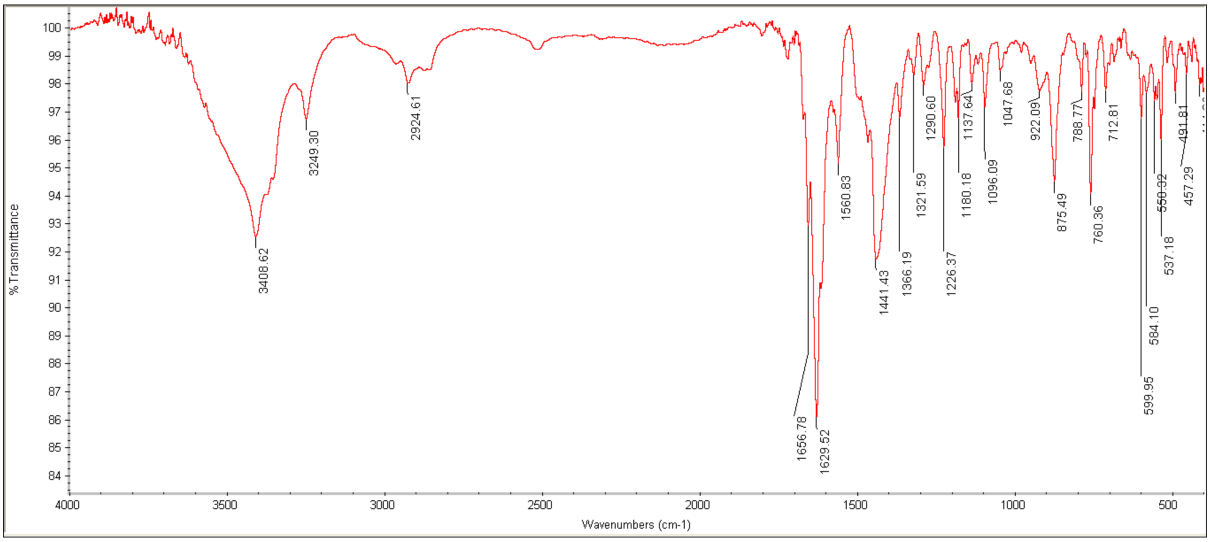

****

***7-(4-chlorophenyl)-10-thioxo-7,10,11,12-tetrahydro-6H-chromeno[3',4':5,6]pyrido[2,3-d]pyrimidine-6,8(9H)-dione* (5d)**

FT-IR (KBr, cm^{-1}): 3395, 3347, 3241, 2931, 1669, 1635, 1609, 1596, 1568, 1489, 1436, 1232, 1197, 881, 757, 554, 536.

^{1}HNMR (DMSO-d_6, 250 MHz) δ (ppm): 5.67 (s, CH-Ar, 1H), 7.12-8.16 (m, ArH, 8H), 7.97 (b, NH, 1H), 12.56 (s, NH, 2H).

^{13}CNMR (DMSO-d_6, 62.5 MHz) δ (ppm): 34.7, 96.4, 97.8, 115.9, 118.7, 124.8, 126.2, 129.7, 129.9, 131.8, 134.1, 138.9, 153.3, 156.8, 164.1, 164.4, 167.4, 174.7.

MS, *m/z*: 409.03 (M^+); Anal. Calcd for $C_{20}H_{12}ClN_3O_3S$: C, 58.61; H, 2.95; N, 10.25; Found: C, 58.55; H, 2.91; N, 10.19.

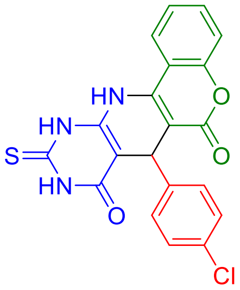

5d

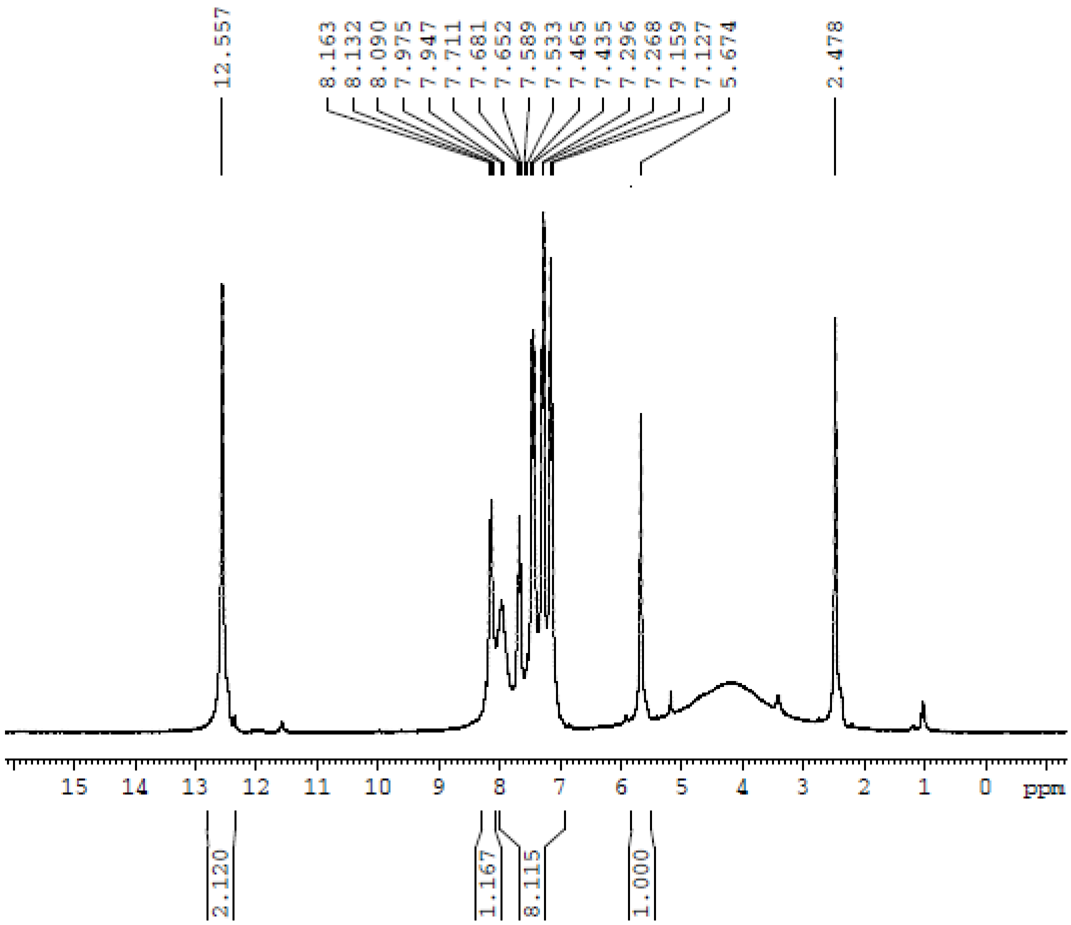
12.557
8.163
8.132
8.090
7.975
7.947
7.711
7.681
7.652
7.589
7.533
7.465
7.435
7.296
7.268
7.159
7.127
5.674
2.478
15 14 13 12 11 10 9 8 7 6 5 4 3 2 1 0 ppm
2.120
1.167
8.115
1.000

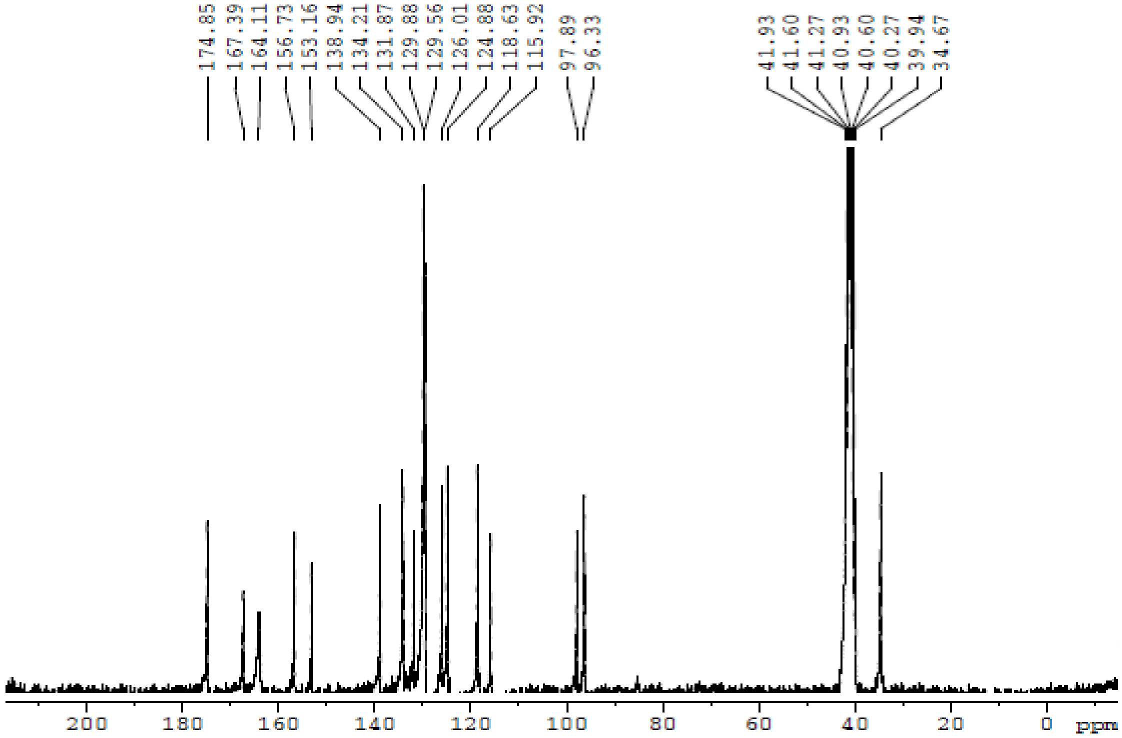
174.85
167.39
164.11
156.73
153.16
138.94
134.21
131.87
129.88
129.56
126.01
124.88
118.63
115.92
97.89
96.33
41.93
41.60
41.27
40.93
40.60
40.27
39.94
34.67
200 180 160 140 120 100 80 60 40 20 0 ppm

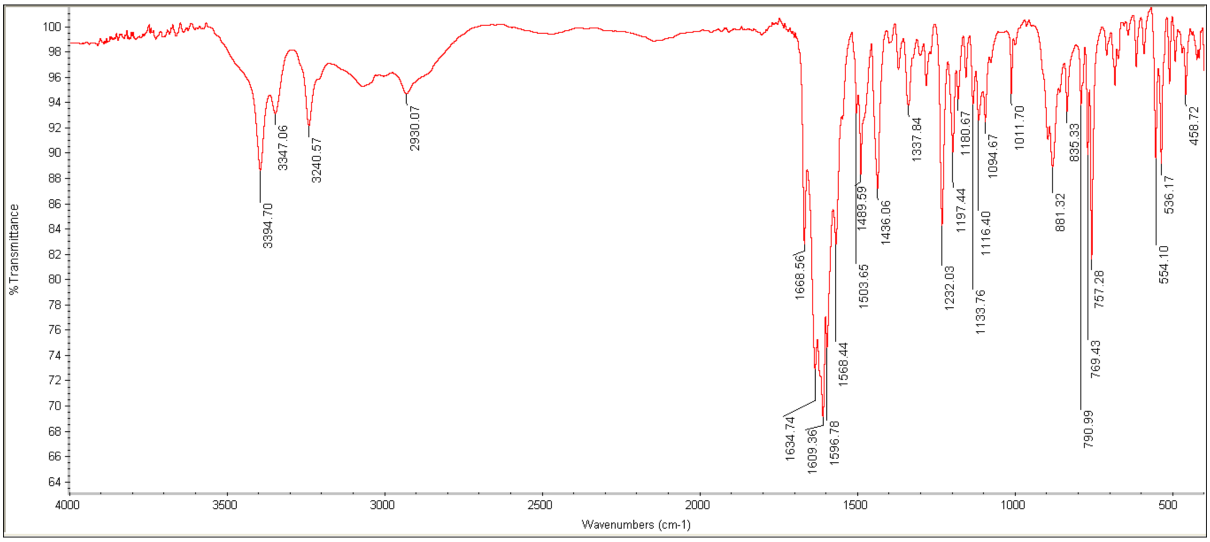

****

***7-(2,4-dichlorophenyl)-10-thioxo-7,10,11,12-tetrahydro-6H-chromeno[3',4':5,6]pyrido[2,3-d]pyrimidine-6,8(9H)-dione* (5e)**

FT-IR (KBr, cm^{-1}): 3417, 3328, 3243, 3061, 1658, 1627, 1613, 1570, 1504, 1435, 1291, 1223, 926, 787, 613.

^{1}HNMR (DMSO-d_6, 250 MHz) δ (ppm): 5.68 (s, CH-Ar, 1H), 7.07-8.09 (m, ArH, 7H), 7.46 (s, NH, 1H), 12.36 (s, NH, 2H).

^{13}CNMR (DMSO-d_6, 62.5 MHz) δ (ppm): 35.14, 95.6, 97.3, 116.0, 118.3, 124.7, 125.6, 128.3, 130.5, 132.2, 132.9, 133.5, 133.8, 134.9, 138.0, 153.2, 155.1, 163.6, 165.8, 174.6

MS, *m/z*:442.99 (M^+); Anal. Calcd for $C_{20}H_{11}Cl_2N_3O_3S$: C, 54.07; H, 2.50; N, 9.46; Found: C, 54.14; H, 2.41; N, 9.55.

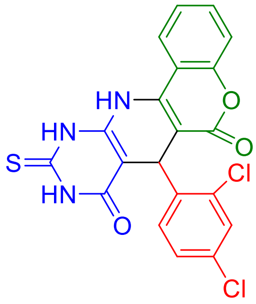

5e

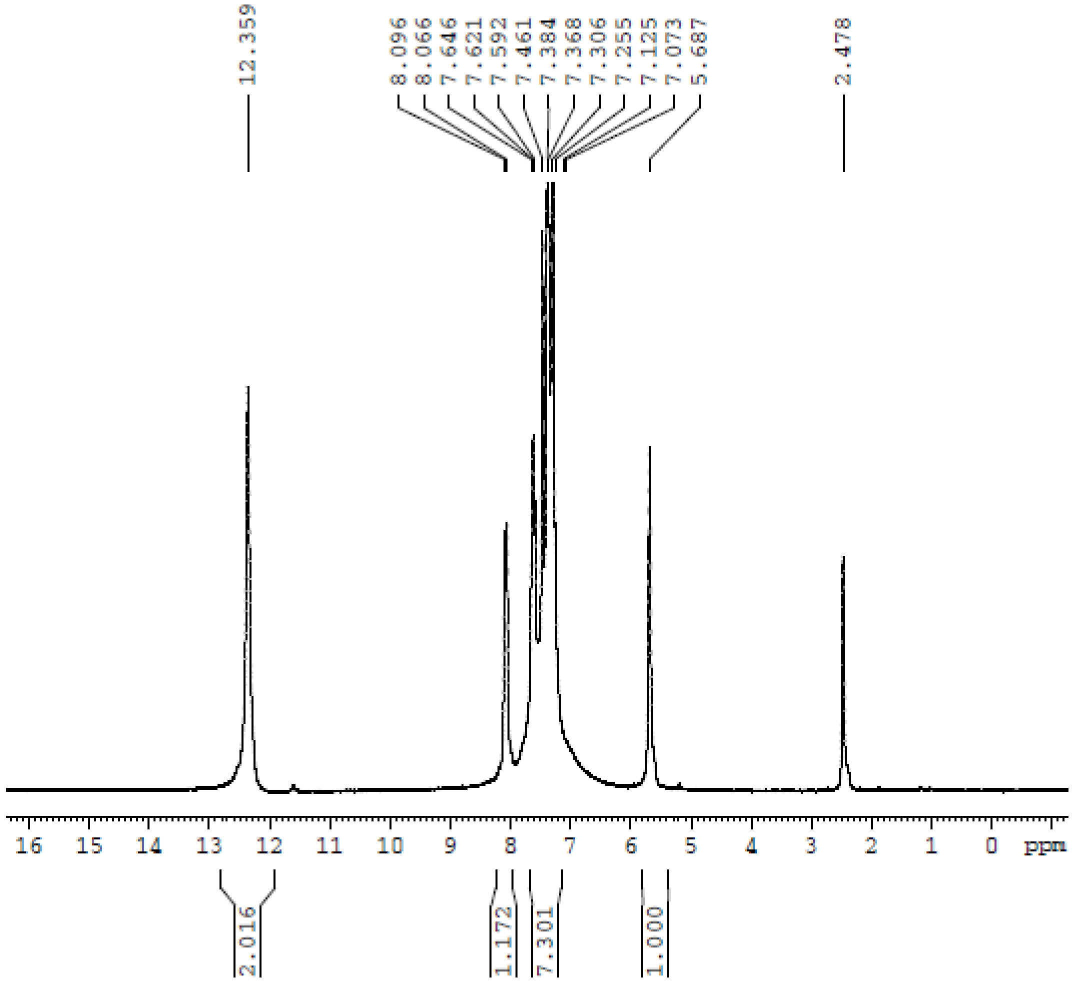
12.359
8.096
8.066
7.646
7.621
7.592
7.461
7.384
7.368
7.306
7.255
7.125
7.073
5.687
2.478
16 15 14 13 12 11 10 9 8 7 6 5 4 3 2 1 0 ppm
2.016
1.172
7.301
1.000

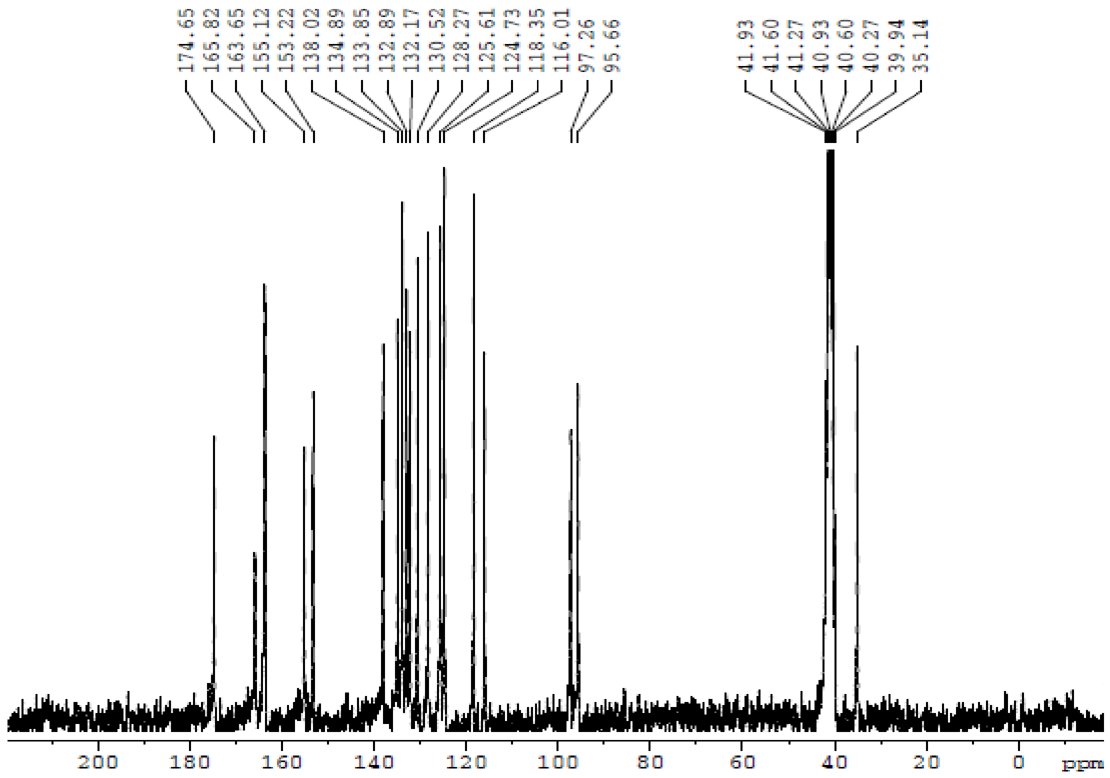
174.65
165.82
163.65
155.12
153.22
138.02
134.89
133.85
132.89
132.17
130.52
128.27
125.61
124.73
118.35
116.01
97.26
95.66
41.93
41.60
41.27
40.93
40.60
40.27
39.94
35.14
200 180 160 140 120 100 80 60 40 20 0 ppm

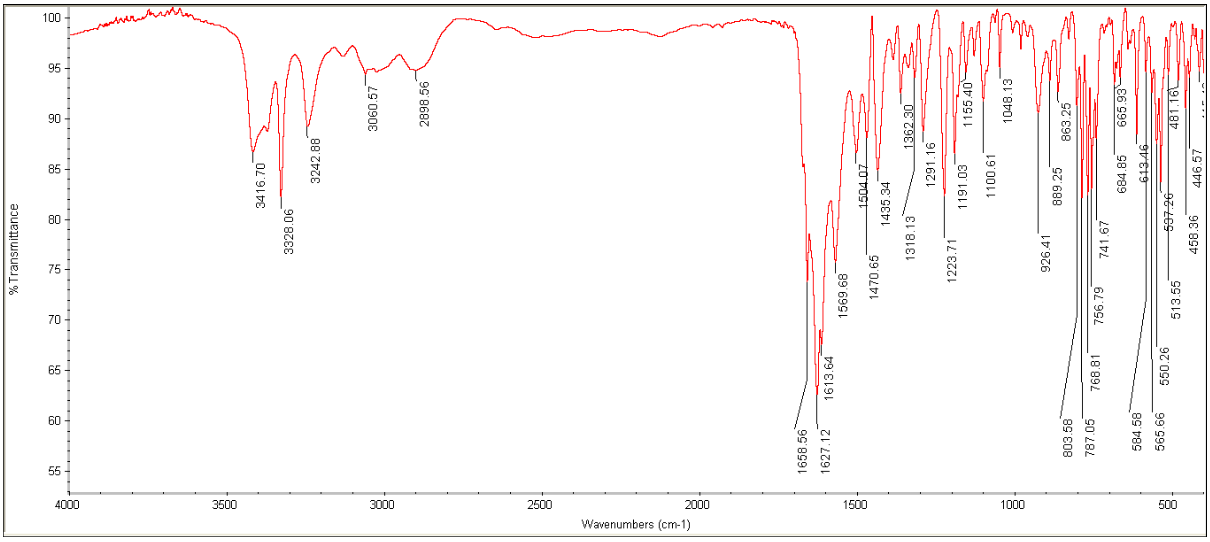

****

***7-(2-nitrophenyl)-10-thioxo-7,10,11,12-tetrahydro-6H-chromeno[3',4':5,6]pyrido[2,3-d]pyrimidine-6,8(9H)-dione* (5f)**

FT-IR (KBr, cm^{-1}): 3412, 3236, 2857, 1658, 1626, 1572, 1522, 1437, 1348, 1224, 1093, 891, 865, 785, 755, 555

^{1}HNMR (DMSO-d_6, 250 MHz) δ (ppm): 6.17 (s, CH-Ar, 1H), 7.39-8.11 (m, ArH, 8H), 7.66 (s, NH, 1H), 12.47 (s, NH, 2H).

^{13}CNMR (DMSO-d_6, 62.5 MHz) δ (ppm): 32.7, 94.6, 97.2, 115.5, 118.4, 124.5, 124.6, 125.6, 125.8, 129.0, 130.5, 133.54, 133.7, 134.3, 150.9, 153.1, 156.3, 164.1, 167.6, 174.8.

MS, *m/z*:420.05 (M^+); Anal. Calcd for $C_{20}H_{12}N_4O_5S$: C, 57.14; H, 2.88; N, 13.33; Found: C, 57.29; H, 2.78; N, 13.28.

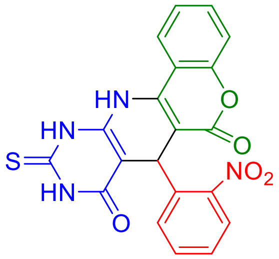

5f

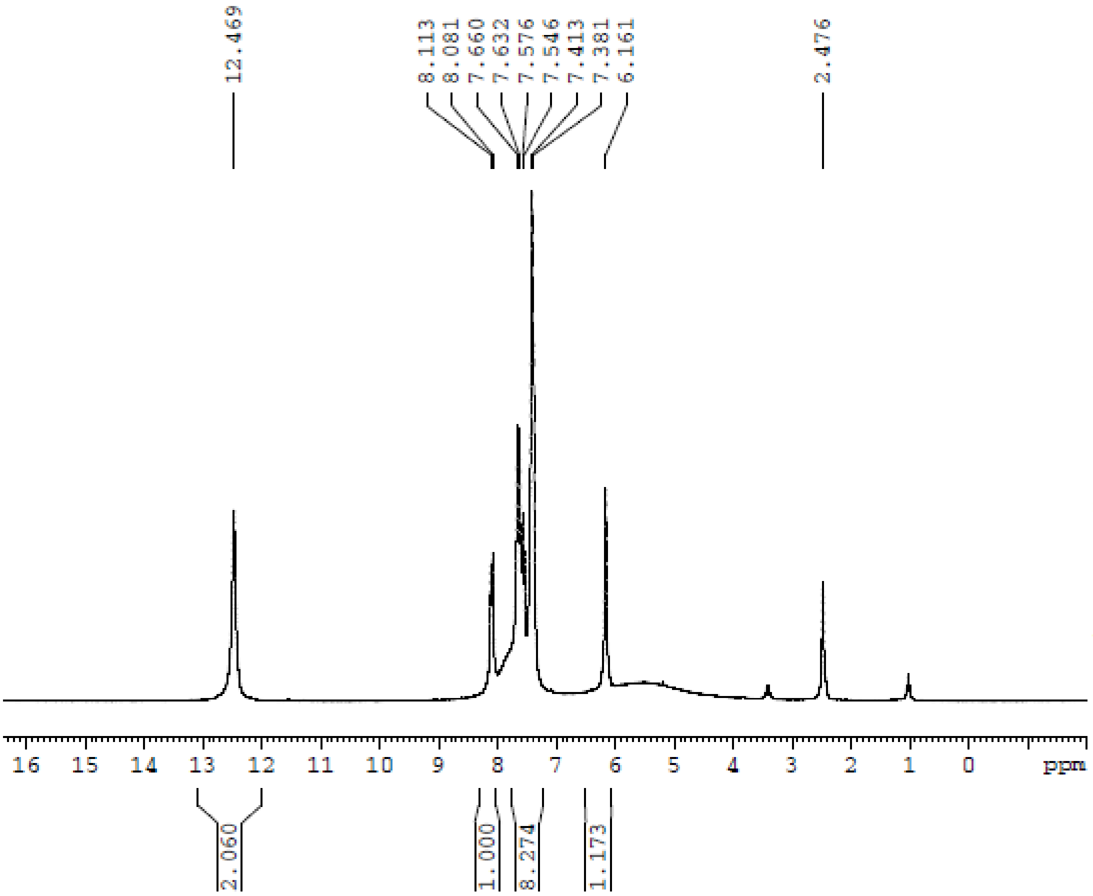
12.469
8.113
8.081
7.660
7.632
7.576
7.546
7.413
7.381
6.161
2.476
16 15 14 13 12 11 10 9 8 7 6 5 4 3 2 1 0 ppm
2.060
1.000
8.274
1.173

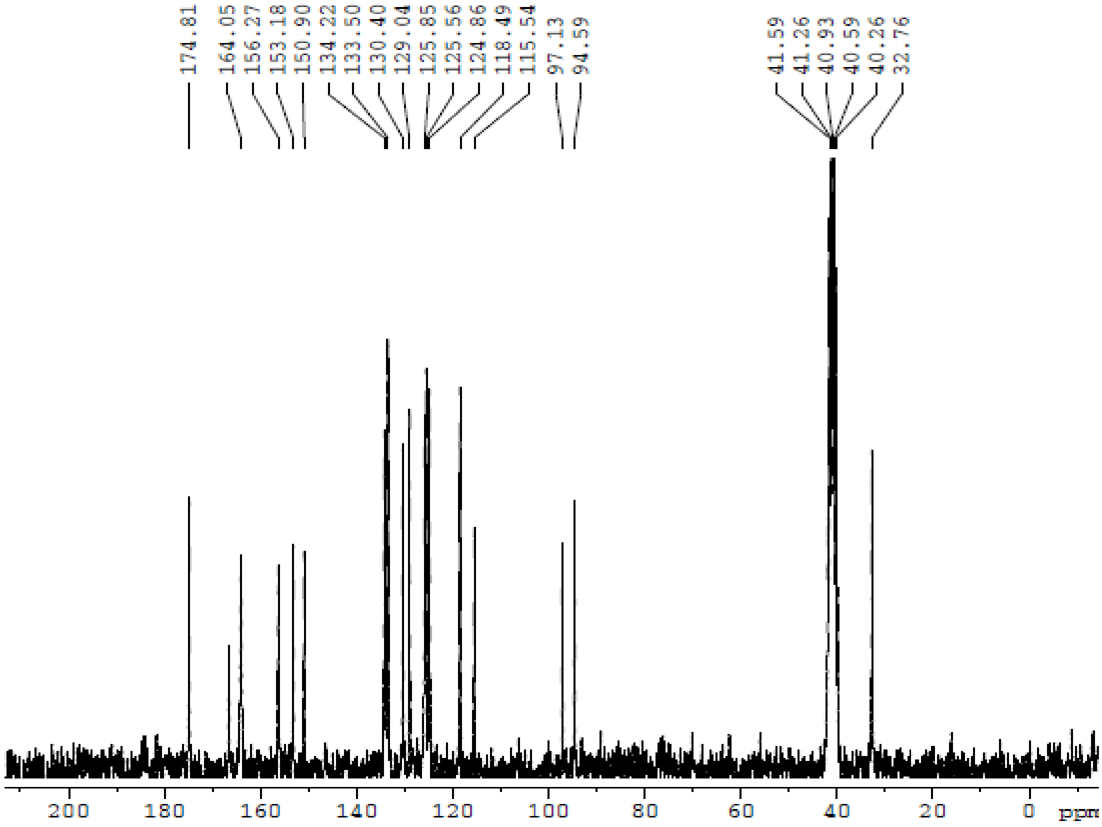
174.81
164.05
156.27
153.18
150.90
134.22
133.50
130.40
129.04
125.85
125.56
124.86
118.49
115.54
97.13
94.59
41.59
41.26
40.93
40.59
40.26
32.76
200 180 160 140 120 100 80 60 40 20 0 ppm

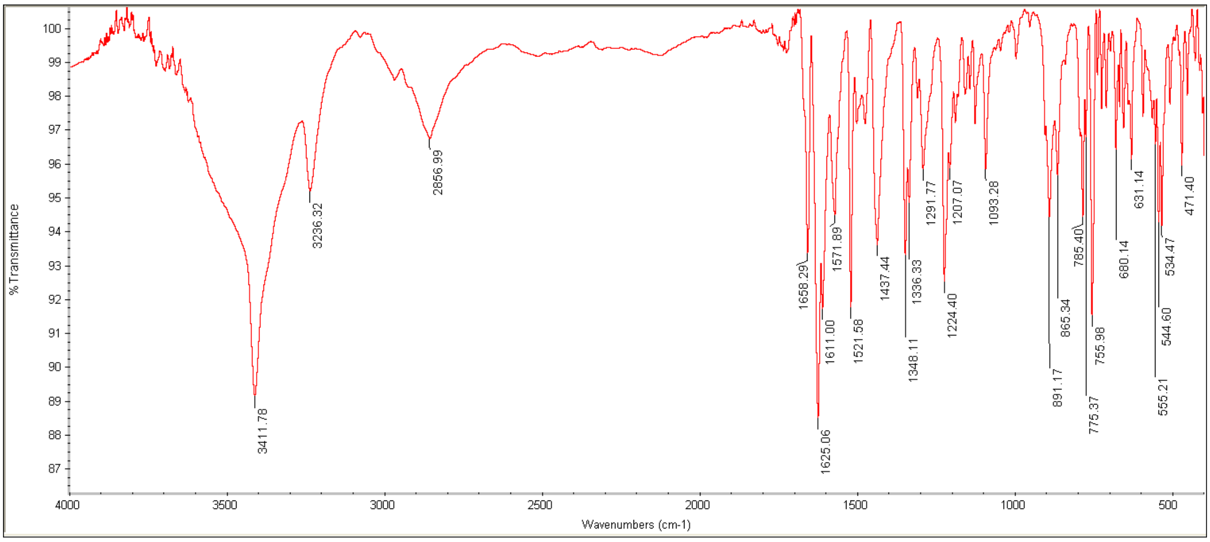

****

7-(4-Nitrophenyl)-10-thioxo-7,10,11,12-tetrahydro-6Hchromeno[3',4':5,6]pyrido[2,3-d]pyrimidine-6,8(9H)-dione (5g)

FT-IR (KBr, cm^{-1}): 3441, 3224, 2923, 1633, 1615, 1515, 1440, 1348, 1210, 1112, 847, 757, 537, 461.

^{1}HNMR (DMSO-d_6, 250 MHz) δ (ppm): 5.82 (s, CH-Ar, 1H); 7.40-8.16 (m,ArH, 8H), 8.10 (s, NH, 1H), 12.52 (s, NH, 2H).

^{13}CNMR (DMSO-d_6, 62.5 MHz) δ (ppm): 35.6, 96.1, 97.4, 115.8, 118.65, 124.7, 124.9, 125.8, 129.5, 130.1, 134.2, 147.2, 148.8, 153.2, 156.7, 164.1, 164.5, 164.7, 167.3, 174.8

MS, *m/z*:420.05 (M^+); Anal. Calcd for $C_{20}H_{12}N_4O_5S$: C, 57.14; H, 2.88; N, 13.33; Found: C, 57.26; H, 2.91; N, 13.39

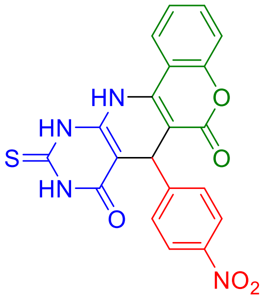

5g

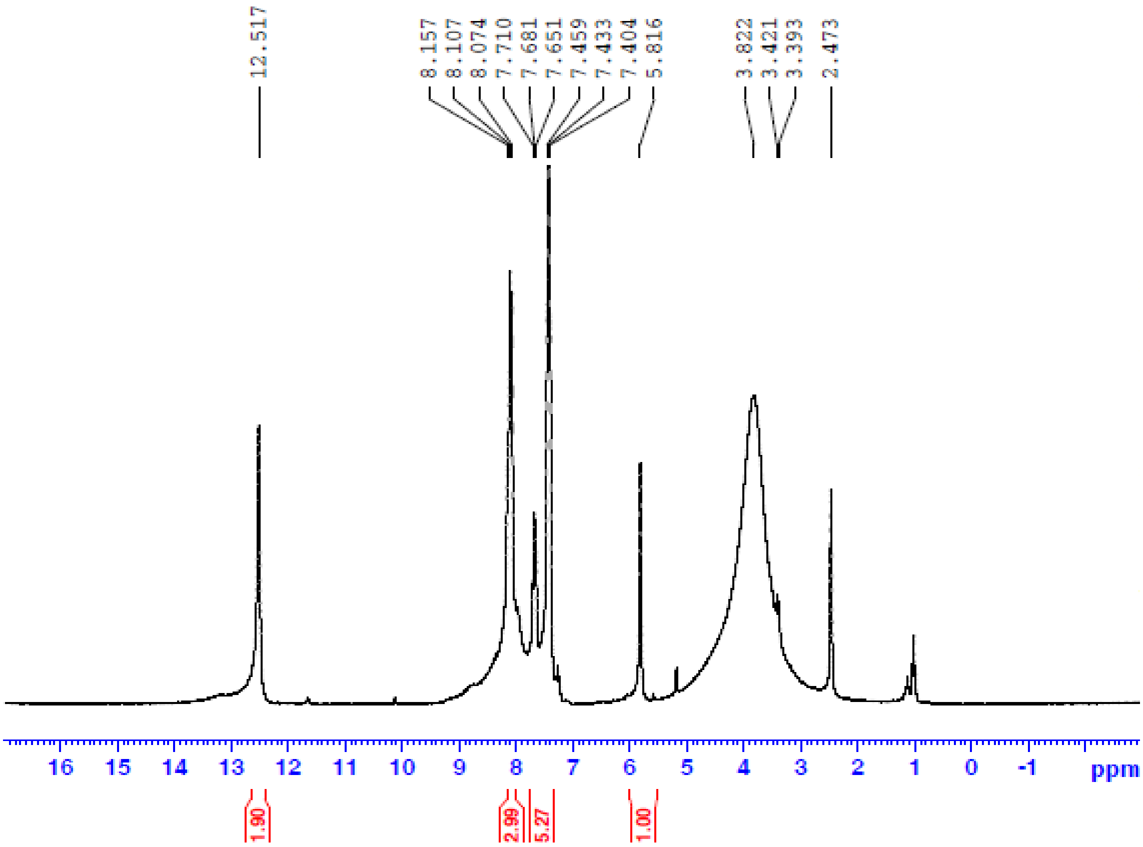
12.517
8.157
8.107
8.074
7.710
7.681
7.651
7.459
7.433
7.404
5.816
3.822
3.421
3.393
2.473
16 15 14 13 12 11 10 9 8 7 6 5 4 3 2 1 0 -1 ppm
1.90
2.99
5.27
1.00

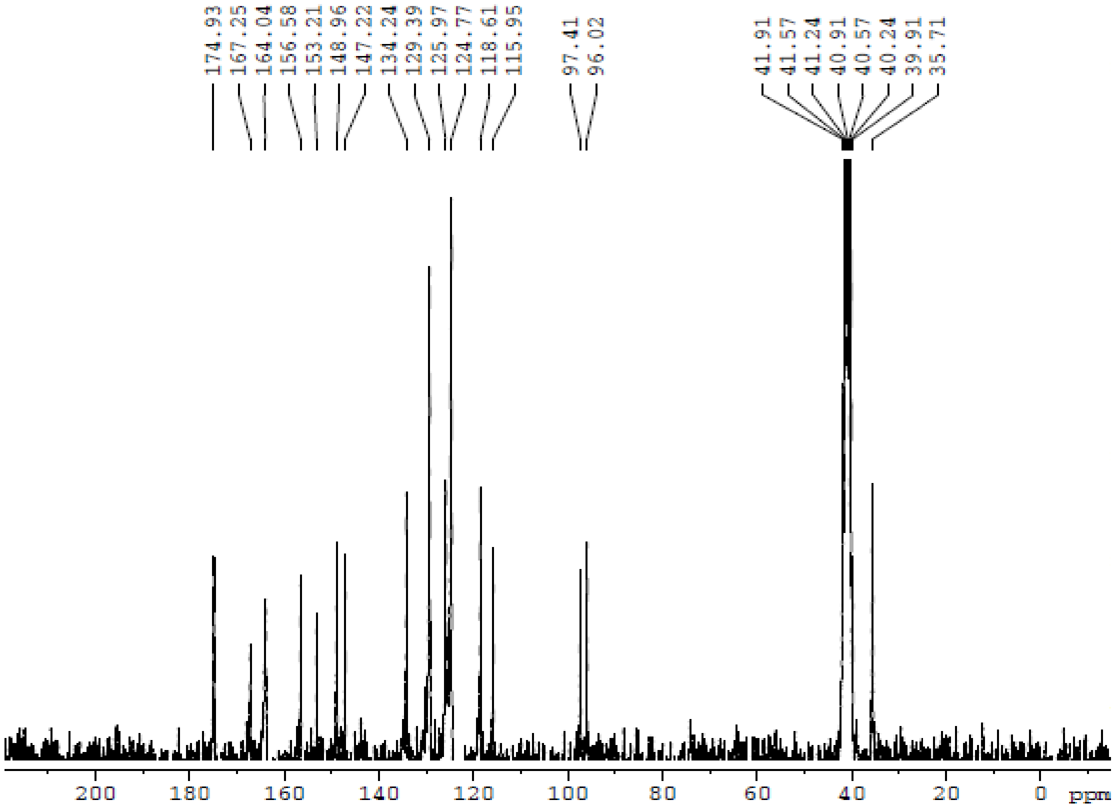
174.93
167.25
164.04
156.58
153.21
148.96
147.22
134.24
129.39
125.97
124.77
118.61
115.95
97.41
96.02
41.91
41.57
41.24
40.91
40.57
40.24
39.91
35.71
200 180 160 140 120 100 80 60 40 20 0 ppm

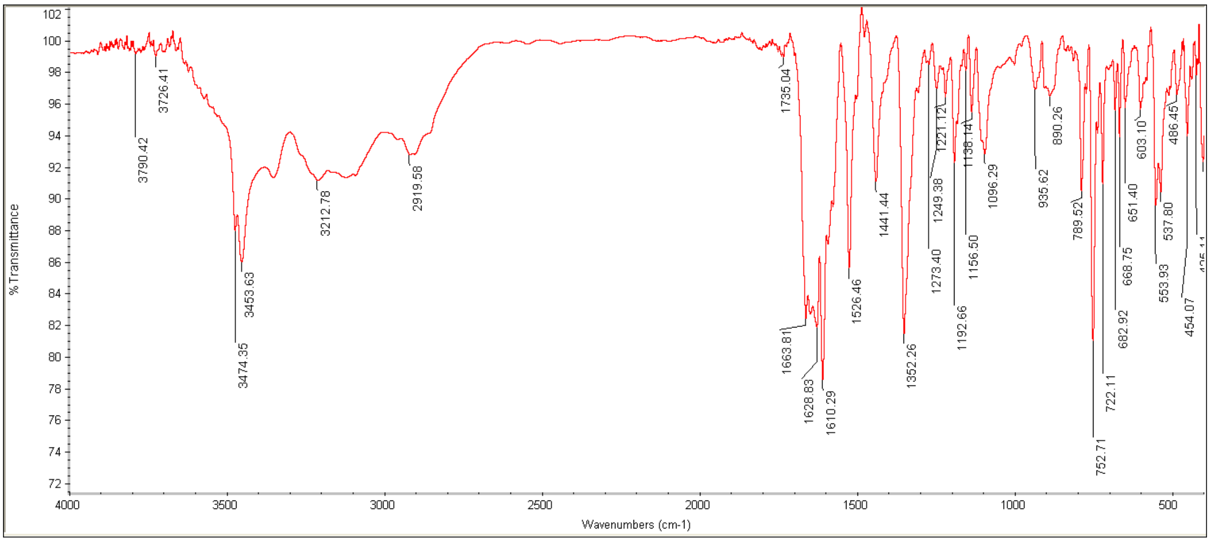

****

***7-(3-methoxyphenyl)-10-thioxo-7,10,11,12-tetrahydro-6H-chromeno[3',4':5,6]pyrido[2,3-d]pyrimidine-6,8(9H)-dione* (5h)**

FT-IR (KBr, cm^{-1}): 3395, 3343, 3239, 1671, 1634, 1609, 1483, 1436, 1368, 1250, 1224, 1130, 1050, 752, 549.

^{1}HNMR (DMSO-d_6, 250 MHz) δ (ppm): 3.65 (s, CH_3, 3H); 5.66 (s, CH-Ar, 1H), 6.61 (s, ArH, 1H), 6.68-8.17 (m, ArH, 7H), 7.95 (b, NH, 1H), 12.62 (s, NH, 2H).

^{13}CNMR (DMSO-d_6, 62.5 MHz) δ (ppm): 34.8, 56.4, 96.4, 98.3, 112.1, 114.3, 115.9, 118.6, 120.3, 124.9, 126.0, 130.7, 134.2, 141.2, 153.3, 156.4, 163.7, 164.1, 164.2, 167.5, 174.8.

MS, *m/z*: 405.08 (M^+); Anal. Calcd for $C_{21}H_{15}N_3O_4S$: C, 62.21; H, 3.73; N, 10.36; Found: C, 62.15; H, 3.79; N, 10.45.

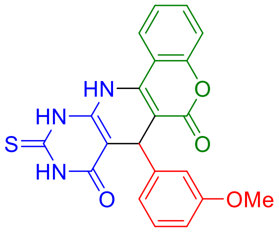

5h

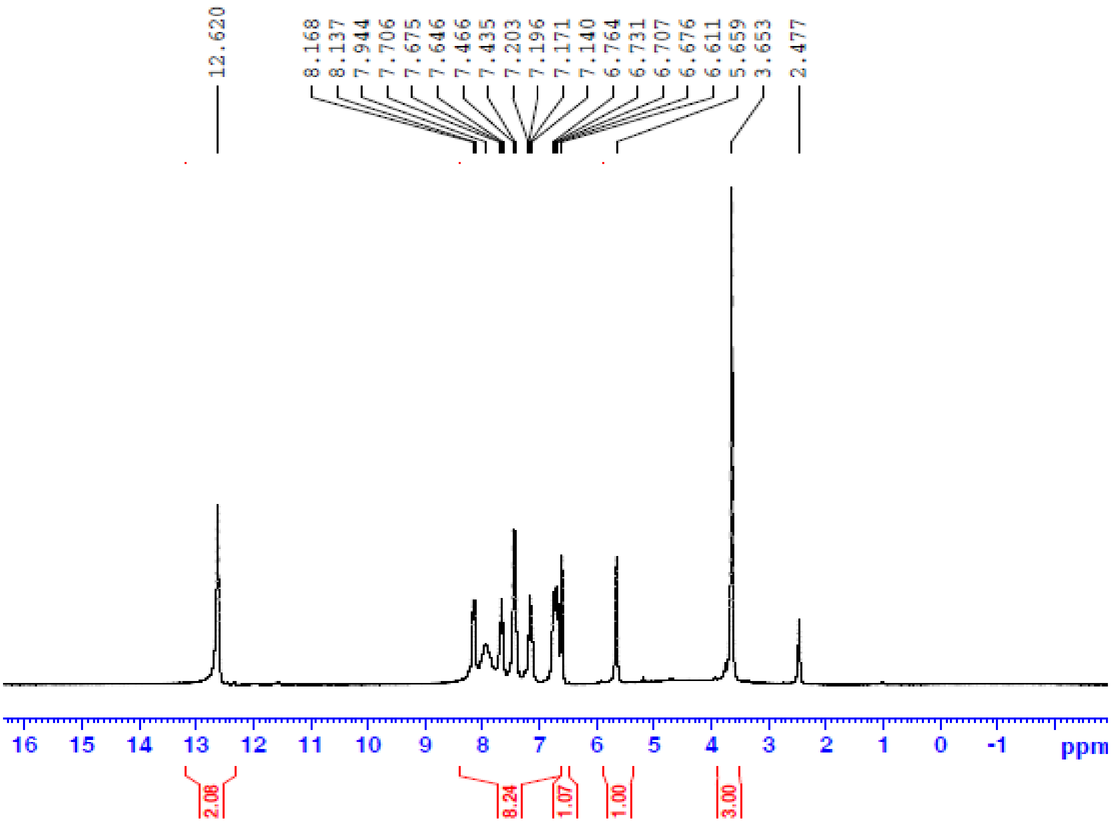
12.620
8.168
8.137
7.944
7.706
7.675
7.646
7.466
7.435
7.203
7.196
7.171
7.140
6.764
6.731
6.707
6.676
6.611
5.659
3.653
2.477
16 15 14 13 12 11 10 9 8 7 6 5 4 3 2 1 0 -1 ppm
2.08
8.24
1.07
1.00
3.00

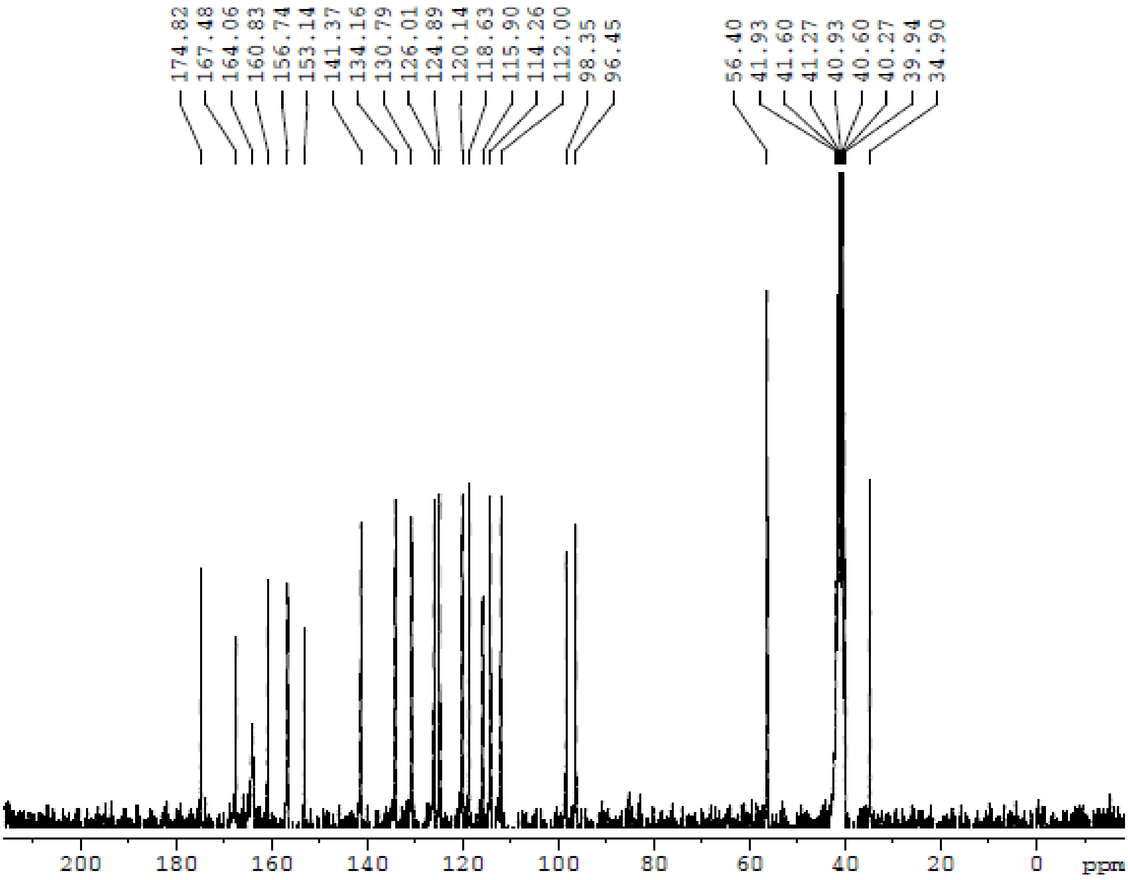
174.82
167.48
164.06
160.83
156.74
153.14
141.37
134.16
130.79
126.01
124.89
120.14
118.63
115.90
114.26
112.00
98.35
96.45
56.40
41.93
41.60
41.27
40.93
40.60
40.27
39.94
34.90
200 180 160 140 120 100 80 60 40 20 0 ppm

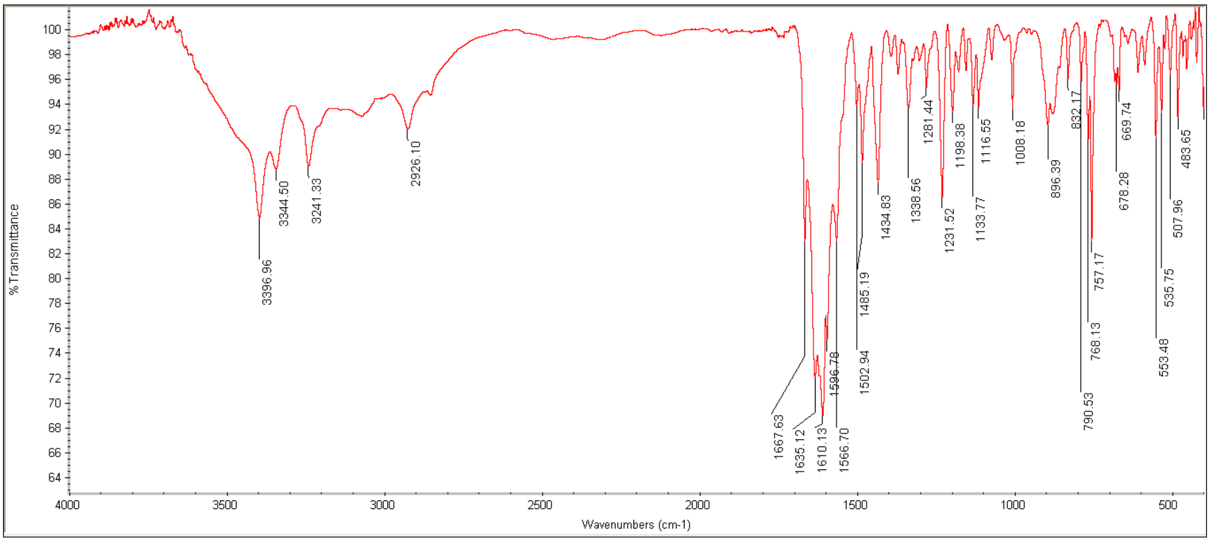

****

4-(6,8-dioxo-10-thioxo-7,8,9,10,11,12-hexahydro-6H-chromeno[3',4':5,6]pyrido[2,3-d]pyrimidin-7-yl)benzonitrile (5i)

FT-IR (KBr, cm^{-1}): 3398, 3345, 3243, 2232, 1668, 1633, 1608, 1567, 1501, 1434, 1230, 1196, 1187, 903, 884, 755, 550.

^{1}HNMR (DMSO-d_6, 250 MHz) δ (ppm): 5.77 (s, CH-Ar, 1H), 7.32-8.15 (m, ArH, 8H), 7.99 (b, NH, 1H), 12.48 (s, NH, 2H).

^{13}CNMR (DMSO-d_6, 62.5 MHz) δ (ppm): 35.7, 95.8, 97.6, 110.1, 115.8, 118.6, 120.4, 124.9, 125.9, 129.3, 133.4, 134.2, 146.6, 153.2, 156.5, 164.2, 164.3, 164.3, 164.8, 167.3, 174.8.

MS, *m/z*: 400.06 (M^+); Anal. Calcd for $C_{21}H_{12}N_4O_3S$: C, 62.99; H, 3.02; N, 13.99; Found: C, 62.84; H, 3.18; N, 13.88

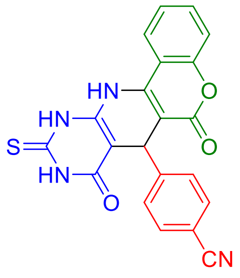

5*i*

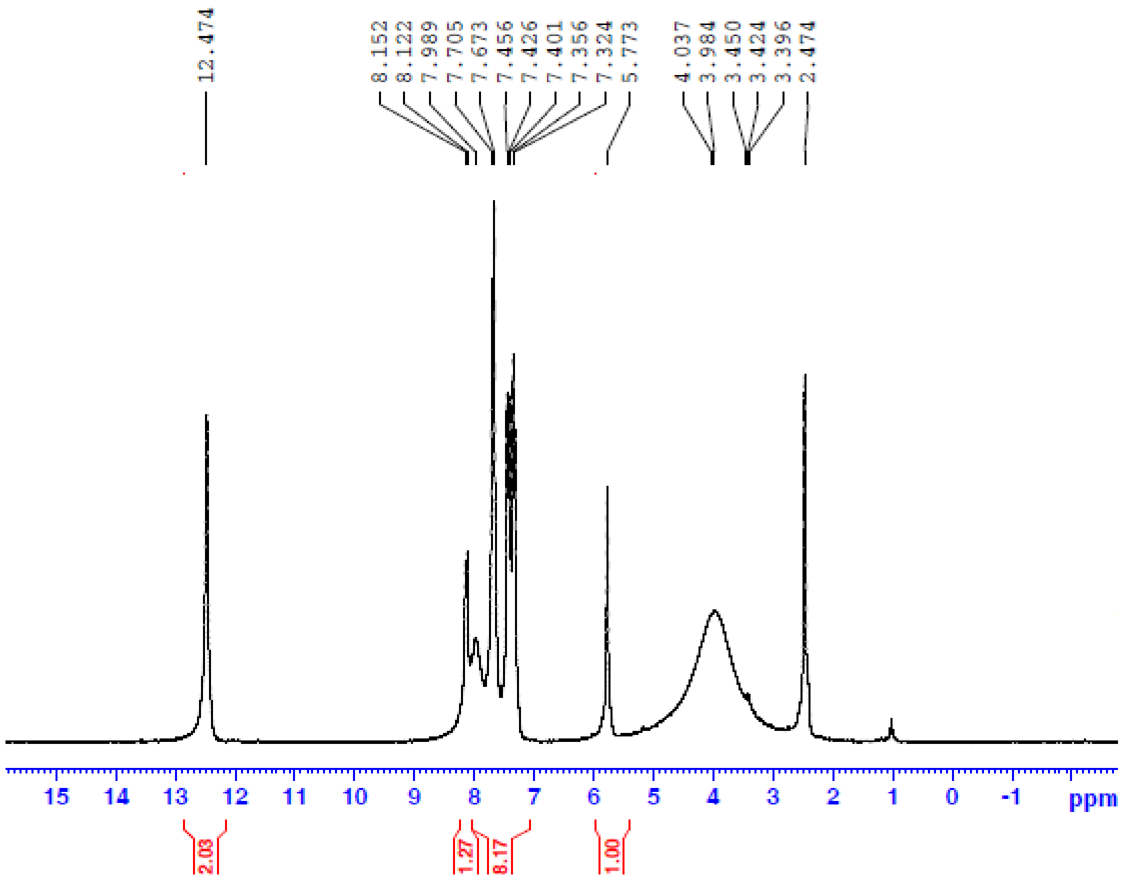
12.474
8.152
8.122
7.989
7.705
7.673
7.456
7.426
7.401
7.356
7.324
5.773
4.037
3.984
3.450
3.424
3.396
2.474
15 14 13 12 11 10 9 8 7 6 5 4 3 2 1 0 -1 ppm
2.03
1.27
8.17
1.00

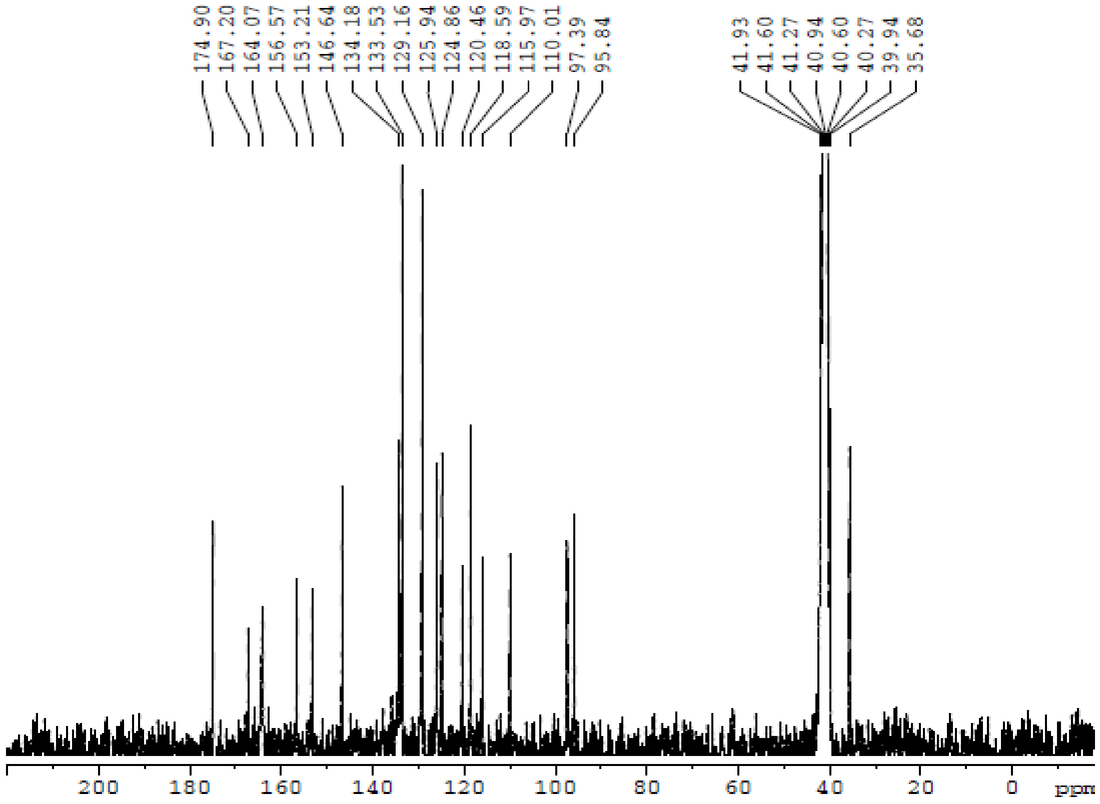
174.90
167.20
164.07
156.57
153.21
146.64
134.18
133.53
129.16
125.94
124.86
120.46
118.59
115.97
110.01
97.39
95.84
41.93
41.60
41.27
40.94
40.60
40.27
39.94
35.68
200 180 160 140 120 100 80 60 40 20 0 ppm

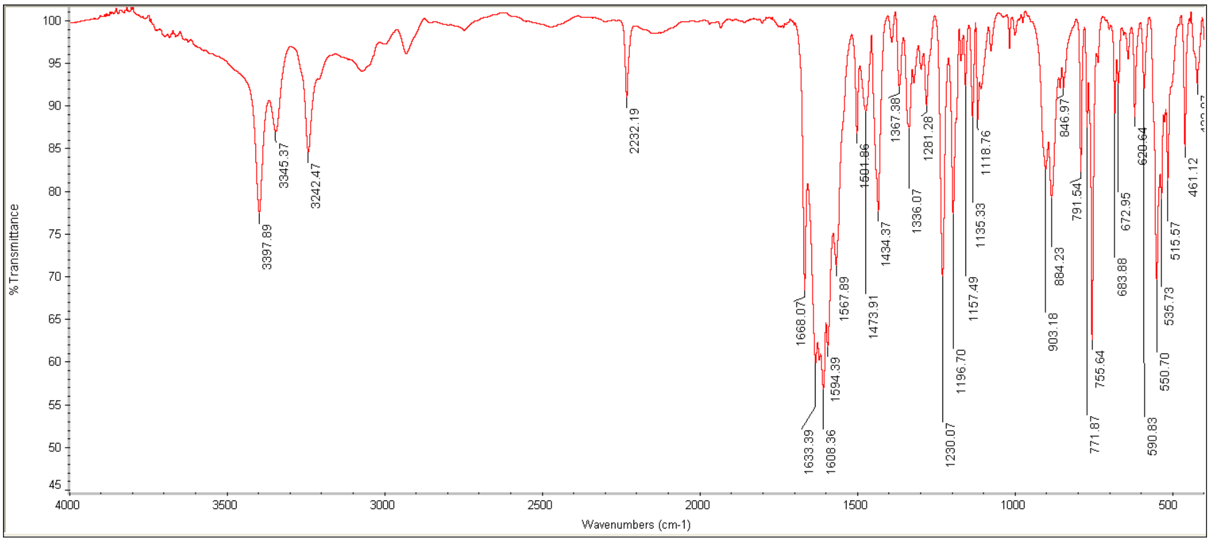

****

7-(4-Bromophenyl)-10-thioxo-7,10,11,12-tetrahydro-6Hchromeno[3',4':5,6]pyrido[2,3-d]pyrimidine-6,8(9H)-dione (5j)

FT-IR (KBr, cm^{-1}): 3397, 3344, 3241, 2926, 1668, 1635, 1611, 1485, 1434, 1338, 1231, 1198, 896, 757, 553, 483.

^{1}HNMR (DMSO-d_6, 250 MHz) δ (ppm): 5.65 (s, CH-Ar, 1H), 7.06-8.15 (m, ArH, 8H), 7.97 (b, NH, 1H), 12.52 (s, NH, 2H).

^{13}CNMR (DMSO-d_6, 62.5 MHz) δ (ppm): 34.9, 96.2, 97.9, 115.7, 118.6, 120.3, 124.8, 125.9, 130.4, 132.4, 132.3 134.3, 139.5, 153.1, 156.7, 164.1, 164.3, 164.9, 167.3, 174.6.

MS, *m/z*: 452.98 (M^+); Anal. Calcd for $C_{20}H_{12}BrN_3O_3S$: C, 52.88; H, 2.66, N, 9.25; Found: C, 52.93; H, 2.72, N, 9.18.

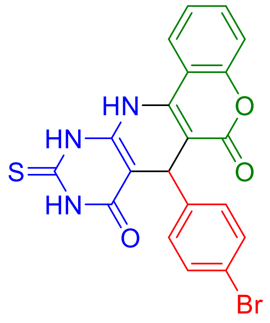

5j

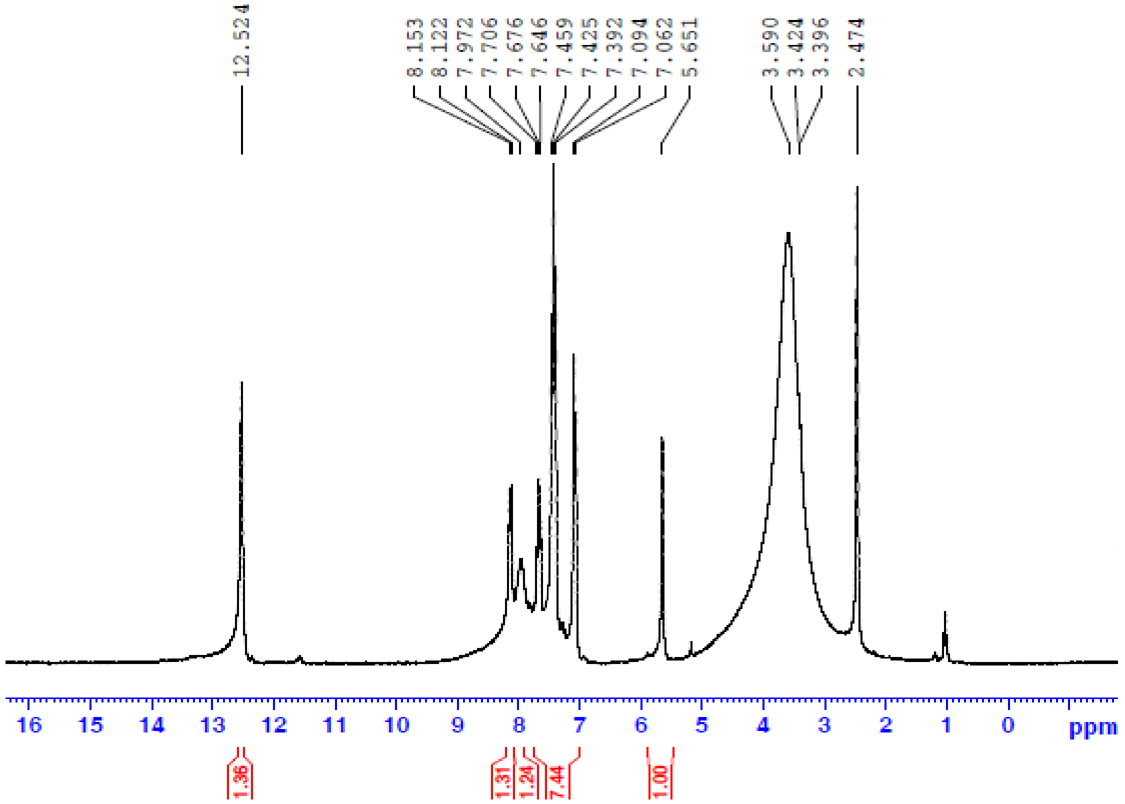
12.524
8.153
8.122
7.972
7.706
7.676
7.646
7.459
7.425
7.392
7.094
7.062
5.651
3.590
3.424
3.396
2.474
16 15 14 13 12 11 10 9 8 7 6 5 4 3 2 1 0 ppm
1.36
1.31
1.24
7.44
1.00

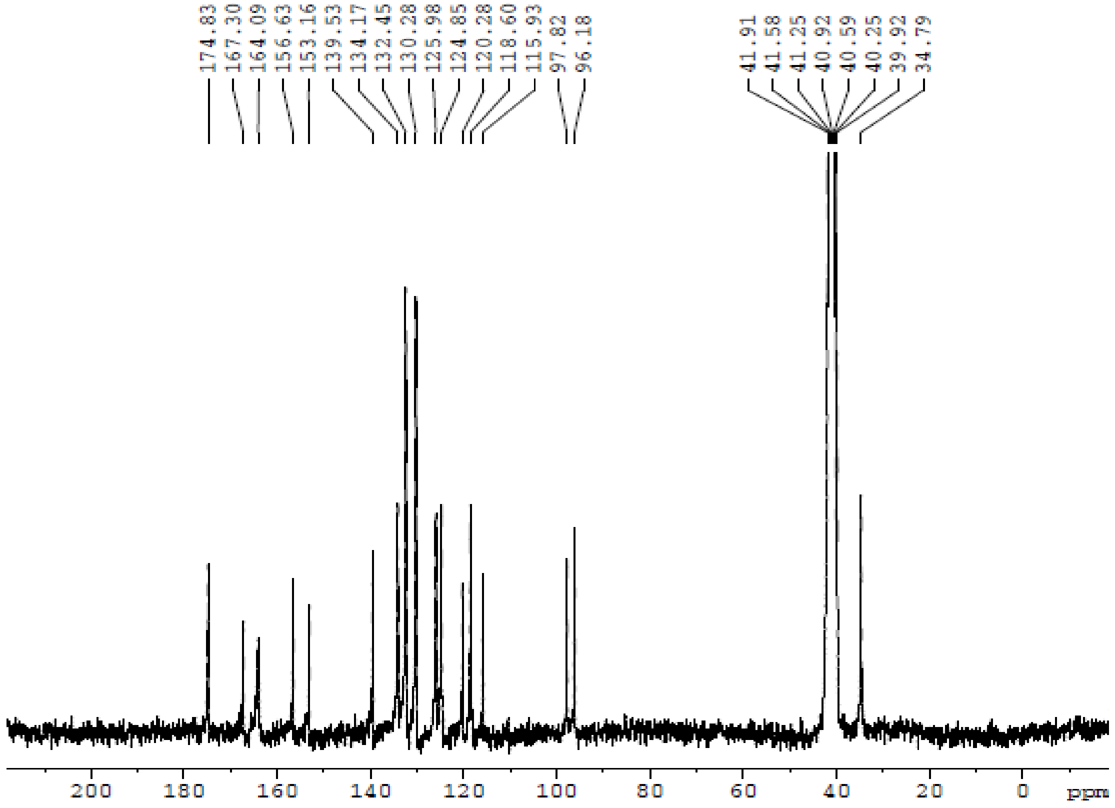
174.83
167.30
164.09
156.63
153.16
139.53
134.17
132.45
130.28
125.98
124.85
120.28
118.60
115.93
97.82
96.18
41.91
41.58
41.25
40.92
40.59
40.25
39.92
34.79
200 180 160 140 120 100 80 60 40 20 0 ppm

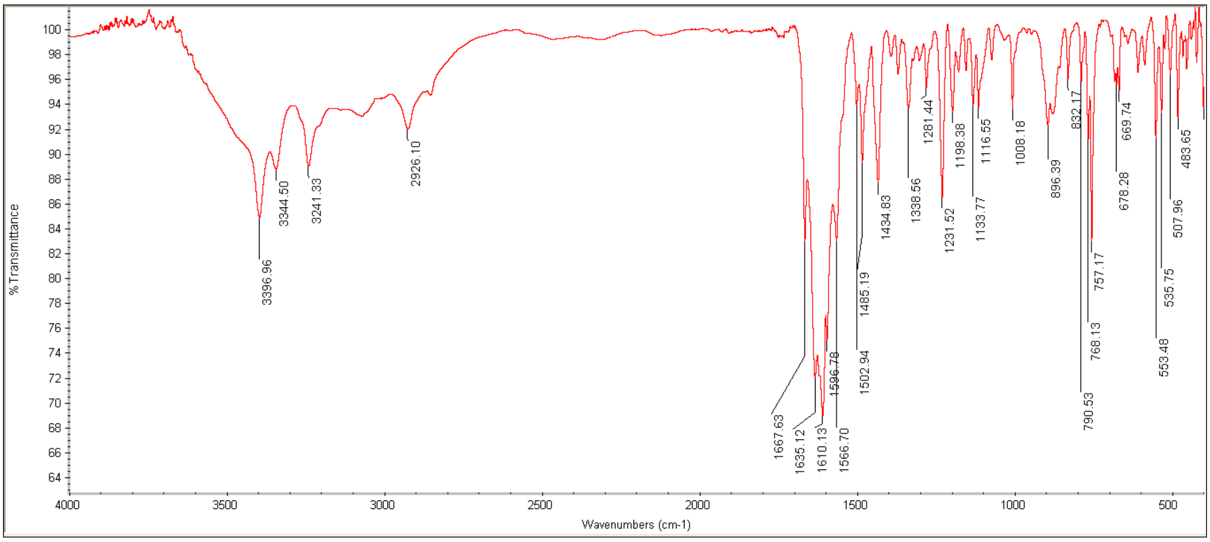

Figure S1. Spectroscopic data and main spectra of produced pyridopyrimidines derivatives (**5a-j**)

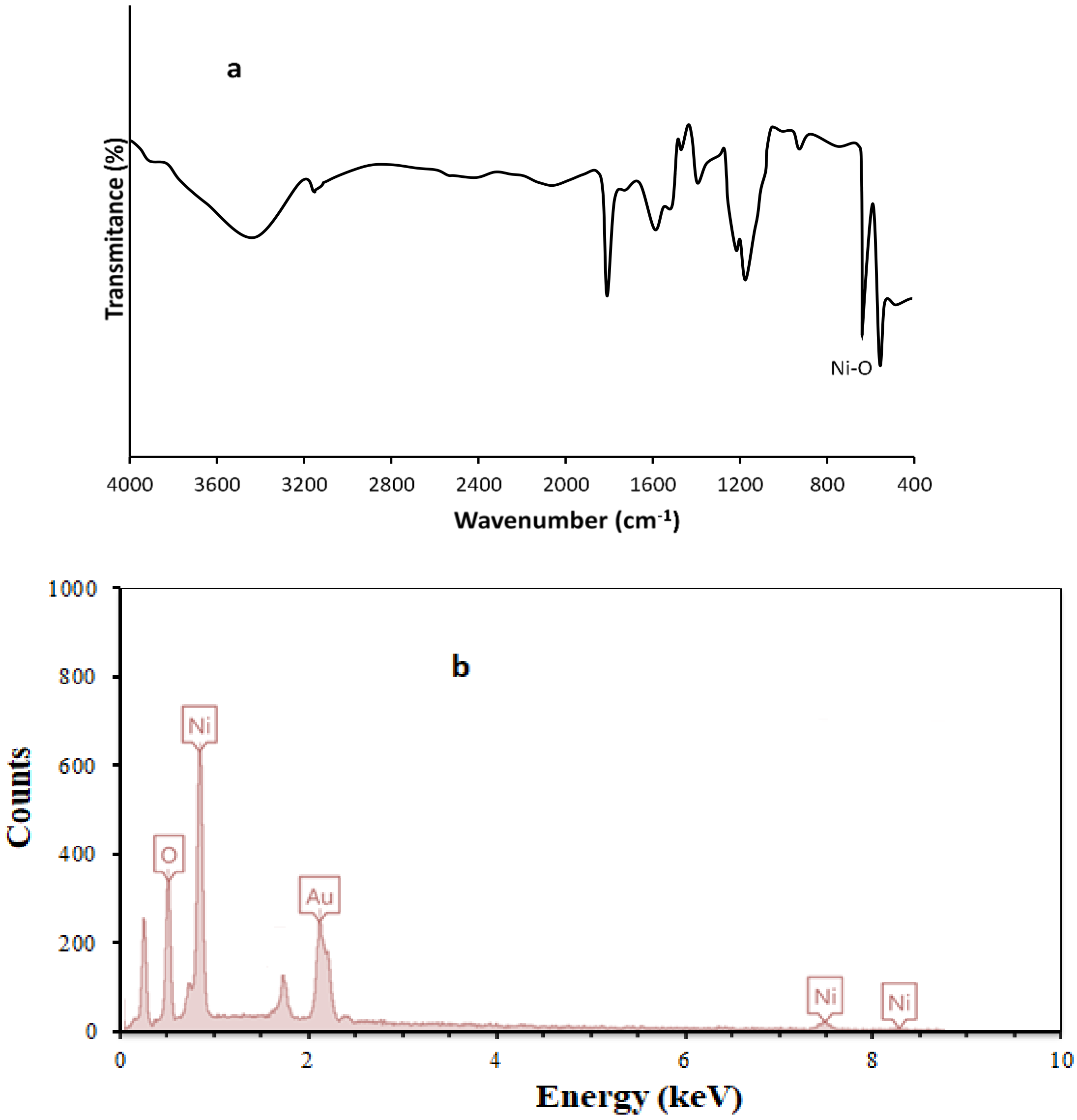

Figure S2. (a) FTIR and (b) EDX spectra of reused biogenic NiO nanocatalyst
